# Transition between cooperative emission regimes in giant perovskite nanocrystals

**DOI:** 10.1038/s41563-026-02544-3

**Published:** 2026-03-31

**Authors:** Etsuki Kobiyama, Gabriele Rainò, Yuliia Berezovska, Chenglian Zhu, Simon C. Boehme, Maryna I. Bodnarchuk, Rainer F. Mahrt, Maksym V. Kovalenko, Thilo Stöferle

**Affiliations:** 1 IBM Research Europe—Zurich, Rüschlikon, Switzerland; 2 Institute of Inorganic Chemistry, Department of Chemistry and Applied Bioscience, ETH Zurich, Zurich, Switzerland; 3 Laboratory of Thin Films and Photovoltaics, Empa—Swiss Federal Laboratories for Materials Science and Technology, Dübendorf, Switzerland

**Keywords:** Nanoparticles, Phase transitions and critical phenomena, Quantum optics, Quantum optics

## Abstract

Interactions between emitters can create cooperative effects that alter light emission. In superfluorescence (SF), excited dipoles couple coherently and radiate collectively, requiring low energetic disorder and strong temporal coherence. Conversely, amplified spontaneous emission results from stimulated amplification and does not require temporal coherence but, unlike SF, sufficient propagation for optical gain. Caesium lead halide perovskite nanocrystals exhibit both amplified spontaneous emission (in disordered films) and SF (in ordered assemblies); however, the connections between these regimes remain unclear. Here we demonstrate that temperature and excitation density can drive the transition between both regimes in a thin film of giant CsPbBr_3_ perovskite nanocrystals. At temperatures below 45 K, excitonic SF was observed, whereas above a transition range between 45 K and 100 K, amplified spontaneous emission prevails but requires increased optical excitation and emitter density. Our results work out the different collective effects present in lead halide perovskites, providing fundamental insights into cooperative phenomena and guidance for the development of compact and bright (quantum) light sources.

## Abstract (web summary)

The role of temperature and excitation density in promoting cooperative emission regimes such as superfluorescence and amplified spontaneous emission, as well as the transitions between such regimes in perovskite nanocrystals, is reported.

## Main

**Fig. 1:**
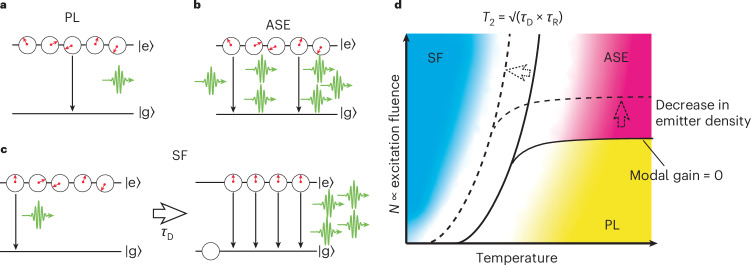
Photon emission processes. **a**–**c**, Illustrations of standard PL (spontaneous emission) (**a**), ASE (**b**) and SF (**c**). The excited and ground states are described as $\mid{\rm{e}}\rangle$ and $\mid{\rm{g}}\rangle$, respectively. The green wavy arrow represents a photon. The white circles represent the emitters with the quantum mechanical phase of the excited electric dipoles as red arrows. *τ*_D_ indicates the time that the emitters need to spontaneously synchronize. **d**, Schematic of the emission regimes. The solid curves correspond to the borders between different emission regimes, and the dashed curves indicate their qualitative shift when the emitter density is decreased.

Lighting and display applications are almost exclusively making use of photoluminescence (PL), where large numbers of emitters radiate light essentially as independent entities (Fig. 1a). Cooperative effects, however, would allow a much higher photon flux and spectrally more brilliant emission. Most prominently, laser devices^1^ deliver coherent emission by placing the optically active material within a resonator in which the light is amplified in an avalanche-like process as it circulates in it. The stimulated emission process that is harnessed in lasers can also be used without a resonator to build sources in which the light output is spectrally much broader and less coherent. In such a waveguiding configuration, a few initial, spontaneously emitted photons become amplified through stimulated emission during their propagation within the excited material. In this amplified spontaneous emission (ASE) process, the photon flux increases exponentially before saturation is reached for a larger propagation length. The effective coupling between the emitters in ASE is induced by propagating photons, whereas the quantum mechanical phases of the emitters do not need to be coherent (Fig. 1b). The ASE emission spectrum typically deviates substantially from the PL spectrum, as the spectral region with the highest net gain is nonlinearly amplified through the stimulated emission process. This yields a substantially narrower emission peak that appears above a threshold on the red side of the PL due to reabsorption.

Alternatively, superfluorescence^2,3^ (SF) can occur when the emitters have synchronized quantum mechanical phases, effectively behaving as one giant emitter. Even if the emitting dipoles are initially excited incoherently, they can spontaneously develop macroscopic coherence through interactions via the common electromagnetic field and collectively emit a burst of correlated photons (Fig. 1c). In contrast to ASE, a sub-wavelength-sized region is theoretically large enough to host this effect, but sufficiently low homogeneous and inhomogeneous broadening and high oscillator strength are necessary. With excitonic SF, the emission spectrum remains very similar to the PL spectrum, whereas its several key characteristics arise in the temporal emission dynamics that scale with the number of coupled excitons *N*, such as a nonlinearly growing emission peak after a build-up time *τ*_D_ that is followed by accelerated decay with lifetime *τ*_R_ and Burnham–Chiao ringing^4,5^ (Supplementary Note 1).

ASE and SF are based on different physical mechanisms, namely, stimulated emission and coherent dipole–dipole coupling between excited emitters, respectively, and transitions between these regimes may occur^6^. Figure 1d illustrates the dominant emission regimes, depending on the excitation density and temperature. SF emission can be observed when the condition for the dephasing time (${T}_{2} > \sqrt{{\tau }_{\mathrm{D}}{\tau }_{\mathrm{R}}}$) is satisfied^7,8^. For materials in which *T*_2_ at low temperatures is on the order of the single-emitter radiative decay time *τ*_0_, the condition is already met for two coupled emitters. With increasing temperature, phonon-induced dephasing reduces *T*_2_ and, therefore, involves increasingly higher excitation strength to maintain SF, consistent with the *N* dependencies of *τ*_R_ and *τ*_D_. Once the SF requirement of slow decoherence cannot be fulfilled any more, the emission occurs as either ASE or standard PL. The threshold from PL to ASE is given when the optical gain accumulated by propagation through the excited material exceeds the optical losses. In the time domain, the transition from PL to ASE is characterized by an abrupt change in the emission lifetime with increasing excitation density, as the stimulated emission rates depend on the photon density. At a given pump fluence, decreasing the density of emitters reduces both number and density of coherently coupled excitons for the SF regime and the modal gain in the ASE, thereby shifting the boundaries (Fig. 1d, dashed lines).

Although ASE does not require coherence among emitters and has been demonstrated in many material classes, SF has only been observed in a few, select systems such as hydrogen fluoride gases^9^, O_2_^−^ centres in KCl crystals^10^, CuCl quantum dots^11^, InGaAs/GaAs multiple quantum wells^12,13^ and nitrogen-vacancy centres in diamond^14^. However, the transition between the two regimes has only been observed in a singular system^6^, and only via temperature-induced dephasing. Recently, lead halide perovskites have attracted enormous attention due to their exceptional optical properties^15–20^ and technological relevance^21,22^. Their low inhomogeneous and homogeneous broadening as well as their high oscillator strength enabled the observation of SF signatures in ordered perovskite nanocrystal (NC) superlattices^23–25^, with superradiant emission ceasing around 100 K (refs. ^25,26^), in films even up to more elevated temperatures^27,28^. In experiments that included photonic resonators or distributed Bragg reflectors, transitions from SF to cavity-enhanced SF^24^ and cooperative exciton–polariton condensation^29^ have been observed, respectively. In different configurations, ASE has been realized in thin films^30–32^, but it has remained an open question whether the very same perovskite material can support both regimes and how transitions may be experimentally induced and observed.

Here we report transitions between the emission regimes of SF, ASE and PL in films of giant CsPbBr_3_ perovskite NCs. The different regimes have been accessed by systematically altering the exciton dephasing time by the sample temperature, the number density of excitons by the used excitation fluence, the density of NCs by the dilution of NC solutions during film preparation and the photon propagation distance through the pumped stripe length. These results provide comprehensive insights into the different emission mechanisms and transitions between them, which are important for the fundamental understanding of cooperative emission processes and the development of compact, ultrabright light sources.

## SF in ensembles of giant NCs at 6 K

**Fig. 2:**
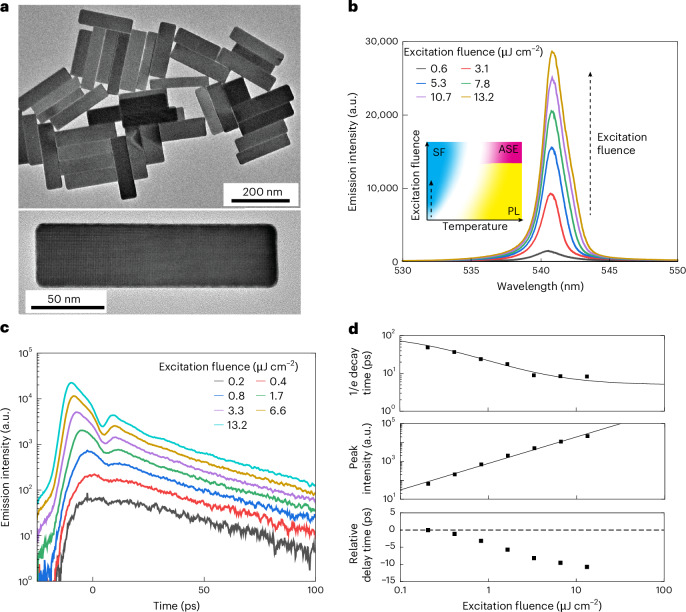
Emission dynamics of the low-density thin film of giant CsPbBr_3_ NCs at 6 K. **a**, Low-resolution (top) and high-resolution (bottom) transmission electron microscopy images of CsPbBr_3_ NCs. The typical particle size of these giant NCs is 40 nm × 40 nm × 200 nm. **b**, Emission spectra for the several excitation fluences at 6 K. In the inset, the arrow indicates the effective trajectory within the emission regime diagram with a change in excitation fluence. **c**, Spectrally integrated emission intensity time traces for several excitation fluences. **d**, Extracted data (squares) as a function of the excitation fluence and the SF model curves (solid lines) of (top) 1/*e* emission decay time, (middle) time-resolved emission peak intensity and (bottom) emission pulse build-up time.

**Extended Data Fig. 1:**
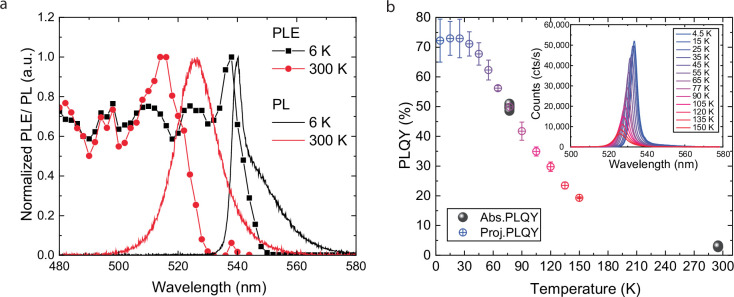
Temperature-dependent emission and absorption of giant CsPbBr3 NCs. **a**, Normalized PL (solid lines) and PLE (solid lines with markers) of the NCs at 6 K (black) and 300 K (red). The detection wavelengths for PLE are 540 nm at 6 K and 524 nm at 300 K. At room temperature, the ensemble shows PL with a single Gaussian emission peak centered at a wavelength of 526 nm and FWHM of 18.1 nm (81 meV), at cryogenic temperature (6 K) the emission red-shifts and narrows down to a Lorentzian shape, the typical spectral shape of a (effective) single dipole, centered at 541 nm with FWHM of 2.8 nm (12 meV). **b**, Temperature dependence of the PL QY of a thin film of giant CsPbBr_3_ NCs. The solid data points show the absolute QY at 77 K and 295 K, obtained via a calibrated absolute-QY spectrometer, and the open circles show the QY at additional temperatures, obtained in a temperature-controlled micro-PL spectrometer and deduced from the relative intensity of the spectrally-integrated PL signal, normalized to the 77 K data point; the error bars represent the standard deviation from measurements taken at four distinct points on the sample, and the inset shows the evolution of the emission spectrum with temperature.

We synthesized CsPbBr_3_ NCs via the ligand-assisted reprecipitation (LARP) technique and prepared drop-cast films on Si/SiO_2_ substrates (Methods). The typical particle size is much larger than the exciton Bohr diameter of ~7 nm (ref. ^21^; Fig. 2a). These ‘giant’ NCs support excitons in the weak-confinement regime with characteristic fine structure and multiexciton states^33^. The excitons in an individual NC are within the Dicke regime^2^ because its volume *V* < *λ*^3^, where *λ* is the emission wavelength. Here we are investigating ensembles and, therefore, cannot fully conclude which of the observations involve inter-NC processes. At 6 K, the PL spectrum has a Lorentzian shape, typical for radiating dipoles (Fig. 2b and Extended Data Fig. 1a). The emission linewidth at cryogenic temperature is much smaller compared with ensembles of NCs in the intermediate-confinement regime due to the lower inhomogeneous broadening of the exciton energies^33^. Moreover, the fact that each NC can host many excitons in contrast to smaller NCs circumvents the dependency on the spatial ordering of NCs that can strongly alter the cooperative emission^25,26^ and would add another, unwanted complexity for this study here. Below 50 K, the quantum yield (QY) exceeds 70%, and hence, the luminescence decay in this temperature range is predominantly radiative (Extended Data Fig. 1b).

First, we study low-density thin films obtained by diluting the NCs in a polymer solution before drop casting (Methods). The emission dynamics is displayed in Fig. 2c. The decay time decreases gradually with increasing excitation fluence, with the 1/*e* emission lifetime (Fig. 2d, top) exhibiting up to tenfold acceleration with respect to the basic emitter decay time of *τ*_0_ = 100 ps (Supplementary Fig. 1a). The almost-linear dependence of the time-integrated emission intensity on the excitation fluence (Supplementary Figs. 2 and 3) shows that this acceleration is not due to quenching by non-radiative decay. The excitation fluence dependence of the peak intensity, *I*_peak_, is best fitted by a nonlinear power-law dependence with an exponent of *α* = 1.4 (Fig. 2d, middle). Although in the Dicke regime, the dependence for SF is theoretically quadratic, extended halide perovskite systems have been observed with *α* = ~1.4–1.7 probably due to saturation effects and photon emission from non-SF domains^23,25,28^ as well as the coupling of emitters beyond the Dicke regime (*V* > *λ*^3^). The build-up time *τ*_D_ shortens as a function of excitation fluence (Fig. 2d, bottom). The above-discussed excitation-fluence-dependent emission dynamics are typical signatures of SF.

## SF-to-PL transition in giant NCs

**Fig. 3:**
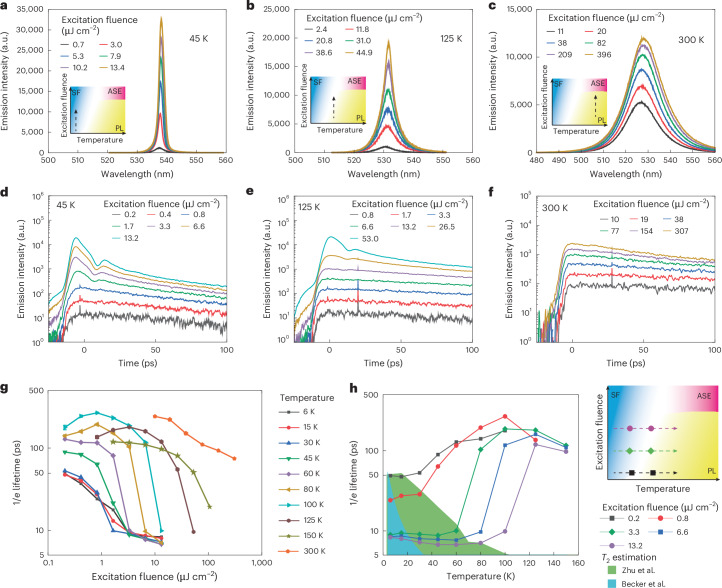
Temperature dependence of emission dynamics of the low-density thin film of giant CsPbBr_3_ NCs. **a**–**c**, Emission spectra for the several excitation fluences at 45 K (**a**), 125 K (**b**) and 300 K (**c**). The inset indicates a change in the main emission regime in the emission regime diagram under the change in excitation fluence. **d**–**f**, Spectrally integrated emission intensity time traces for the several excitation fluences at 45 K (**d**), 125 K (**e**) and 300 K (**f**). **g**, 1/*e* emission decay time as a function of excitation fluence at different temperatures. **h**, 1/*e* emission decay time as a function of temperature for several excitation fluences. The green- and blue-shaded areas indicate the estimated exciton dephasing time *T*_2_. For the estimation, we have taken the reported *T*_2_ value of smaller CsPbBr_3_ NCs at 4 K from ref. ^15^ as a reference. The temperature dependencies were estimated based on the evolution of the emission linewidth of NCs^38^ (green trace, labelled as Zhu et al.) and on four-wave-mixing measurements with CsPbBr_2_Cl perovskite NCs^16^ (blue trace, labelled as Becker et al.).

Since faster phonon-induced dephasing at increasing temperatures may render it increasingly more difficult to build up a collective SF state, we investigate the anticipated transition from SF to standard PL or ASE^6,34^. The time-integrated emission spectra (Fig. 3a–c and Supplementary Figs. 2 and 3) do not exhibit any sign of an (abrupt) transition between regimes. Except for 300 K, the spectra are fit well by a single Lorentzian peak. The emission peak shifts towards higher energy, and its width increases with increasing temperature (Supplementary Figs. 4 and 5). The peak energy shift is consistent with the temperature dependence of the bandgap energy of halide perovskites^35,36^, and spectral broadening arises from coupling to thermally excited phonons, indicating decreasing coherence.

In contrast to the time-integrated emission spectra, the time-resolved emission (Fig. 3d–f and Supplementary Fig. 6) shows a pronounced transition in the emission dynamics with temperature. Below 45 K, the emission is consistent with SF, inferred from the accelerated decay with increasing excitation fluence and the observation of Burnham–Chiao ringing. For temperatures between 45 K and 150 K, a rather abrupt acceleration of decay occurs above a threshold at increasingly higher fluences. In particular, the approximately constant 1/*e* decay time from PL at a lower fluence suddenly drops to the same SF-accelerated decay time as that at low temperature.

Quantitative analyses of emission dynamics also indicate the change in emission regime (Fig. 3g and Supplementary Fig. 7). The black curves in Supplementary Fig. 7 represent the fitting results based on a model function, which comprises a constant decay for the PL regime and a threshold above which the SF-accelerated decay regime shows its characteristic fluence dependence (Methods). The peak intensities *I*_peak_ follow a power law with exponents *α* = 1.4–1.8, except for 300 K. Remarkably, they do not exhibit a threshold-like change, as would be expected from the transition between PL (*α* = 1) and SF (*α* = 2). Such a superlinear growth of *I*_peak_ in the PL regime could be a consequence of gradual filling of below-bandgap trap states^37^ with increasing excitation fluences, given the non-unity PL QY at high temperature (Extended Data Fig. 1b). In addition, the experimental data of the pulse build-up time, below 100 K, shows the typical trend expected for SF: the emission occurs earlier with increasing fluence. Above 100 K, no fluence dependence is observed for the build-up time.

Figure 3h shows the temperature dependence of the decay time for different fluences. We attribute the observed behaviour to the decrease in dephasing time *T*_2_ with temperature; therefore, the SF condition time ${T}_{2} > \sqrt{{\tau }_{\mathrm{D}}{\tau }_{\mathrm{R}}}$ requires a larger number of excited emitters *N* to be fulfilled, because *τ*_R_ ∝ *τ*_0_/*N*. In these perovskites, *T*_2_ is comparable with *τ*_0_ at low temperatures^15^, and therefore, SF starts already from the lowest excitation fluence, whereas at elevated temperatures, an increasingly higher excitation fluence is required for a transition from PL to SF (Fig. 3a,b, insets). Hence, although at low temperature, SF-accelerated decay occurs without a pronounced threshold because the low dephasing does not prevent collective emission even for weak excitation, at intermediate temperatures, a threshold develops because a certain *N* is needed to realize a cooperative state with sufficiently fast decay that is faster than *T*_2_. The trend for the transition between PL and SF is qualitatively similar to the expectation from the *T*_2_ time (Fig. 3h, shaded region), whereas quantitatively, there can be differences depending on how the *T*_2_ estimate is obtained^16,38^ (Fig. 3h). At room temperature (Fig. 3c), even at very high excitation fluence (>300 µJ cm^−2^), no SF signatures were observed.

## SF-to-ASE transition in dense NC ensembles

**Fig. 4:**
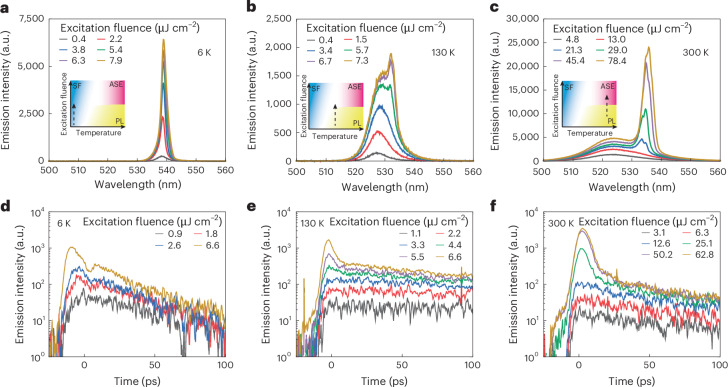
Temperature dependence of emission dynamics of the high-density thin film of giant CsPbBr_3_ NCs. **a**–**c**, Emission spectra for several excitation fluences at 6 K (**a**), 130 K (**b**) and 300 K (**c**). The inset indicates the sampled regimes in the emission regime diagram with the change in excitation fluence. **d**–**f**, Spectrally integrated emission intensity time traces for different excitation fluences at 6 K (**d**), 130 K (**e**) and 300 K (**f**).

**Extended Data Fig. 2:**
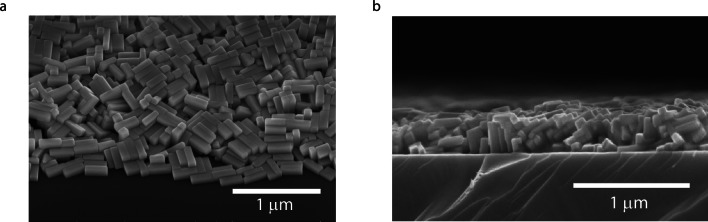
Scanning electron microscopy (SEM) image of the high-density thin film. **a**, Tilted top-view image of the NC film. **b**, Cross-section image of the NC film from a cleaved edge of the substrate.

**Extended Data Fig. 3:**
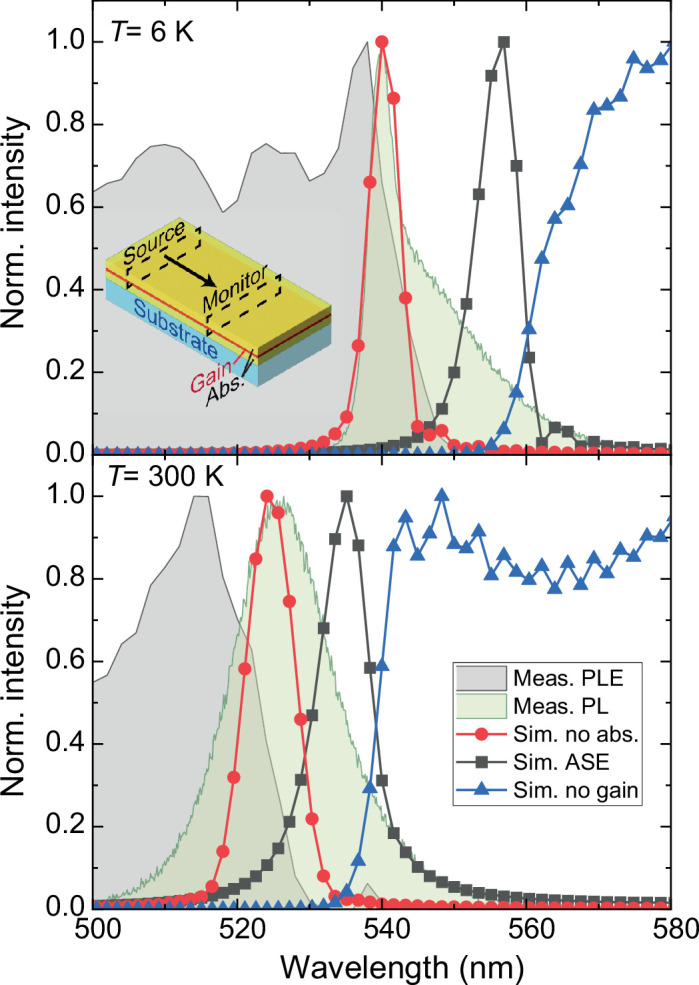
Simulated ASE spectra at 6 K and 300 K. The measured PLE (grey) and PL (light green) spectra, which are identical to Extended Data Fig. 1 and re-plotted here for direct reference, are used as inputs for the FDTD simulation as material absorption and gain spectra, modelling them via phenomenological multi-oscillator fits with positive and negative imaginary refractive index, respectively. The inset illustrates the simulation geometry where a waveguide on a substrate is realized that supports a single vertical guided mode that is launched by a broadband source (effectively ‘white’ over the wavelength range) into the waveguide and then spectrally monitored after several micrometers of propagation. The perovskite material is artificially divided into a thin gain layer (labelled ‘Gain’) sandwiched between two absorbing layers (labelled ‘Abs.’) that are modelled with the experimentally obtained respective spectra. This effectively enables one to simulate distinct absorption and gain spectra that both affect the same propagating mode and are characteristic of a non-fully inverted emitter population. The red curves show the simulated spectra at the ‘Monitor’ position in the absence of absorption, that is, where the imaginary refractive index of the two ‘Abs.’ layers is set to zero, resulting in nonlinearly amplified PL spectra with peak positions coinciding with those of the PL spectra but with reduced widths due to gain narrowing. The black curves show the simulated ASE spectra at the ‘Monitor’ position, where both absorption and gain are included in the simulation by their respective imaginary index dispersions, leading to narrow ASE emission redshifted by ~15 nm or 65 meV, irrespective of the temperature. The blue curves show the simulated spectra at the ‘Monitor’ position, where the imaginary index of the ‘Gain’ layer is set to zero, effectively showing white-light transmission spectra with a reduced blue part due to material absorption.

Next, we study high-density thin films of NCs obtained by drop casting polymer-free solutions of NC (Methods and Extended Data Fig. 2). The layer thickness and effective refractive index is sufficiently high to allow the guiding of light in the NC layer. Time-integrated PL spectra of these films (Fig. 4a–c and Supplementary Fig. 8) show that below 45 K, the single-peak structure persists with increasing excitation fluence, whereas above 45 K, first a shoulder and then a clearly defined new peak appears above a certain threshold excitation fluence. This emerging peak exhibits strong superlinear growth above the threshold (Supplementary Fig. 9). It is redshifted by up to 10 meV until ~86 K, but then at higher temperatures, by up to 35–45 meV (Supplementary Fig. 10), larger than any trion or biexciton binding energy^33^. It remains narrow at around 10 meV over the whole temperature range (Supplementary Fig. 11). A substantial redshift and narrowing is typical for ASE because due to the small Stokes shift, the peak centre of the excitonic emission is reabsorbed, and optical gain is the highest at wavelengths red-detuned from the PL maximum. Similar energy redshifts and narrowing in the ASE regime can be re-enacted with numerical finite-difference time-domain simulations (Extended Data Fig. 3), showing that narrowing and redshift of tens of millielectronvolts is a robust characteristic of ASE, independent of temperature.

The temporal dynamics (Fig. 4d–f and Supplementary Figs. 12 and 13) at 6 K are qualitatively similar to the low-density sample, with a gradual emission acceleration under increasing fluence, consistent with SF. Comparable acceleration at comparable excitation fluence may suggest that the coupling of excitons within individual NCs would contribute more strongly to SF acceleration than the coupling between various NCs that occurs on top of it, which, however, would need to be confirmed through single-NC experiments. For a quantitative analysis, we use the same fit model as for the low-density sample, as it can capture the threshold behaviour reasonably well. Starting at ~44 K, the development of a threshold for accelerated decay is observed for both low- and high-density samples.

**Extended Data Fig. 4:**
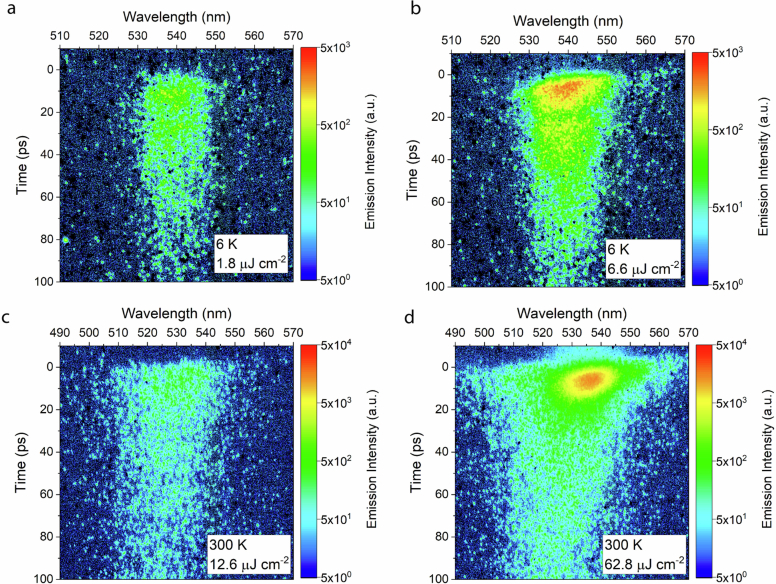
Streak camera emission images from the high-density thin film of giant CsPbBr3 NCs. The sample temperatures are 6 K (**a, b**) and 300 K (**c, d**). The excitation fluences are 1.8 µJ cm^−2^ (**a**), 6.6 µJ cm^−2^ (**b**), 12.6 µJ cm^−2^ (**c**) and 62.8 µJ cm^−2^ (**d**).

**Extended Data Fig. 5:**
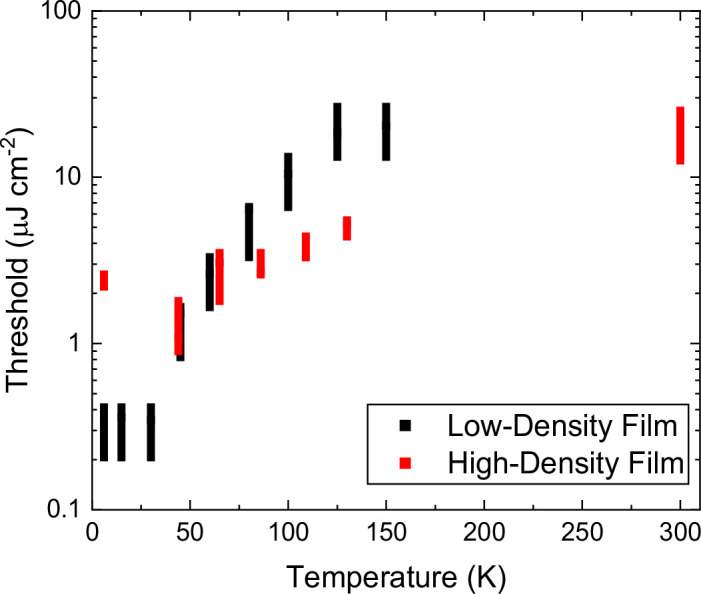
Thresholds for 1/e decay time shortening. The data bars are the threshold obtained for the piecewise fit of the 1/e decay of both the low-density (black) and the high-density (red) sample. The bars denote the fluence range in which the empirical fit model found the transition between the PL and the accelerated SF/ASE regime, given by the fluence values of the data points in the respective 1/e decay curves in Fig. S7 and S13 that are adjacent to the threshold.

However, two important differences arise above the threshold, indicating that the emission from the high-density sample evolves towards the ASE, whereas SF may still coexist at intermediate temperatures. First, as discussed above, there is a concomitant change in the spectrum in which a redshifted, narrow peak in the time-integrated spectra appears suddenly above the threshold. Below 90 K, its energy shift remains within the red-peak’s linewidth, which is an indication for a mixture or crossover of SF and ASE above the threshold. Above this temperature, its redshift exceeds its linewidth, becoming consistent with the behaviour expected from the ASE simulations (Extended Data Fig. 3). Besides, streak camera measurements (Extended Data Fig. 4) indicate that the accelerated emission in the time traces corresponds to this redshifted emission peak in the PL spectra. Second, the subsequent growth of the time-resolved peak intensity *I*_peak_ occurs with a strongly superlinear power-law exponent *α* > 2 (Supplementary Fig. 13), whereas the exponent in the regime below the threshold is only slightly superlinear (*α* = 1.0–1.6), similar to the low-density sample. This concurs with the temperature at which the redshifted shoulder in the time-integrated spectra appears. Presumably, the temperature-induced dephasing increasingly impedes the establishment of SF, favouring ASE at higher temperatures. This would be in line with the evolution of the thresholds (Extended Data Fig. 5), showing that although the threshold for the low-density sample increases by 20 times between 45 K and 130 K, in the high-density sample, it is only 4 times in the same temperature range. This difference is probably attributable to the strong temperature dependence of SF as it is sensitive to emitter decoherence, whereas ASE is not. Although for InGaAs quantum wells, a transition from ASE to SF has been reported at cryogenic temperatures^39^, we have not observed such transition in our measurements.

The different characteristics of dense and diluted films at intermediate temperatures can be understood by considering that SF and ASE are competing processes. In the high-density film, the excitation density is much higher than in the low-density film, and scattering losses in the densely packed film are lower. Therefore, in the low-density film, the net modal gain within the pump spot and the number of PL seed photons is insufficient for a stimulated emission avalanche, and hence, the excitons have time to synchronize spontaneously via coherent interactions and decay cooperatively via SF. Although at higher temperatures at which dephasing is faster, a transition to ASE could nevertheless be possible at an even higher excitation fluence; we did not observe ASE from the low-density film within the excitation fluence range of our experiments. In comparison, in the high-density sample, the higher exciton density increases the optical gain, effectively favouring ASE already at lower temperatures and excitation fluences. As a result, the excitons decay via ASE before forming SF states, which is consistent with vanishing ringing that is observed above ~86 K.

## Experiments and simulations of ASE with variable excitation stripe lengths

**Fig. 5:**
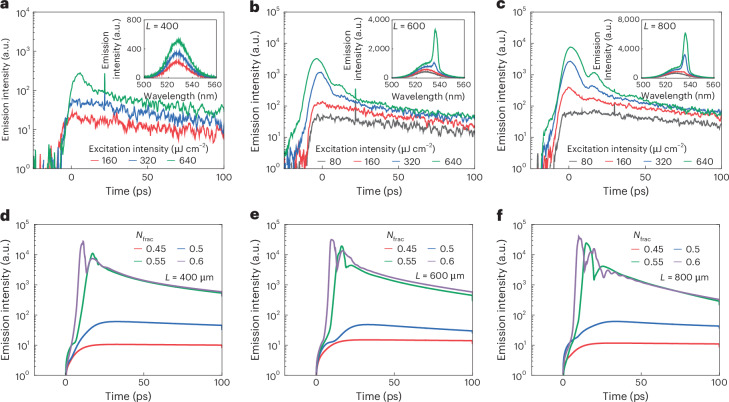
Emission dynamics under variable stripe length excitation at 300 K. **a**–**c**, Spectrally integrated time-resolved emission intensity traces detected from the edge of the sample for several excitation fluences with excitation stripe lengths of 400 µm (**a**), 600 µm (**b**) and 800 µm (**c**). The insets show the corresponding time-integrated emission spectra. **d**–**f**, Numerically simulated emission intensity time traces for different excitation stripe lengths of 400 µm (**d**), 600 µm (**e**) and 800 µm (**f**). The total number density of the emitters in the system was set as *N*_total_ = 10^18 ^cm^−3^. *N*_frac_ indicates the fraction of the excited emitters to *N*_total_ and does not scale linearly with the excitation fluence due to nonlinear processes such as absorption saturation. The simulation model and parameters are discussed in Methods.

**Extended Data Fig. 6:**
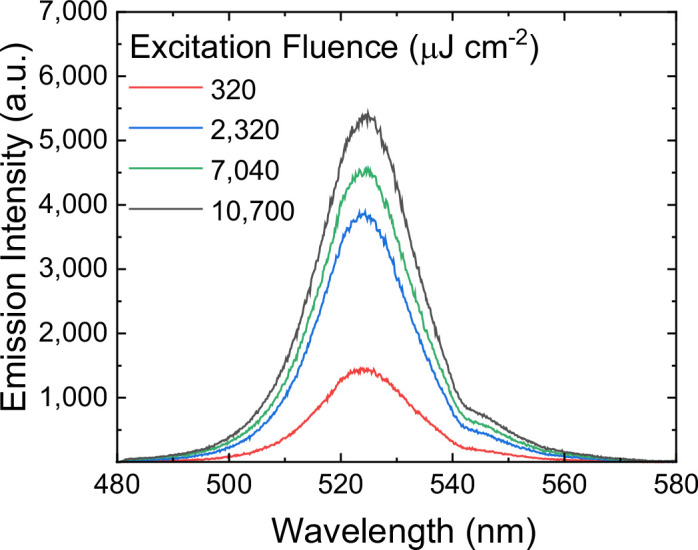
Room-temperature emission spectra from the high-density thin film of giant CsPbBr3 NCs under increasing excitation fluence with a small excitation spot size. The excitation laser pulse was focused on the sample with a 100x objective lens, and the excitation spot size was 3.5 μm FWHM.

**Extended Data Fig. 7:**
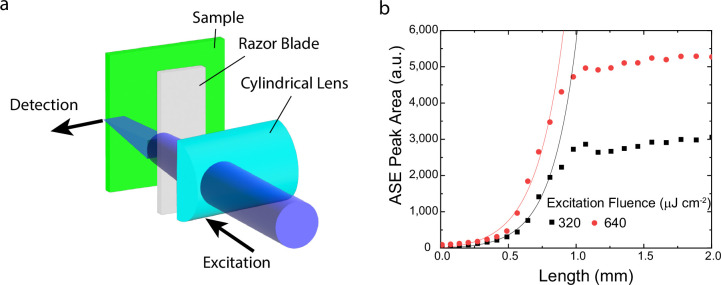
Variable-stripe-length excitation measurements. **a**, Schematic illustration of the experimental setup. The excitation stripe length was controlled by moving the razor blade. **b**, ASE intensity as a function of excitation stripe length at 300 K for two excitation fluences. The data points show the emission peak intensity collected from the sample’s edge. The solid curves are the fitting analysis results with the model function (*I*_ASE_ ∝ (e^*gL*^ – 1)/*g*), where *L* is the excitation stripe length and *g* is modal gain. Fitting the ASE peak area with the given function, yields a modal gain of *g* ~ 51 ± 2 cm^−1^. This is lower compared to values for similar CsPbBr_3_ materials reported in the literature^30^^,49^, but reasonable because the drop-casted thin film is very rough (Extended Data Fig. 2) and therefore, scattering losses are expected to be high.

Interestingly, with a much smaller excitation spot (Methods), no redshifted peak was observed, even up to much higher fluences at room temperature (Extended Data Fig. 6). In contrast to SF, which needs only (sub)wavelength-sized volumes, a substantial pumped gain length is required for ASE. To assess the effect of propagation length on the emission dynamics, we performed variable stripe length measurements^40,41^ (Methods and Extended Data Fig. 7). As shown in Fig. 5a–c (insets), the redshifted emission peak appears at weaker excitation fluence for longer excitation stripe length *L*. In terms of the temporal emission dynamics, fast decay components are more pronounced with larger *L* (Fig. 5a–c), in line with a higher overall gain.

What is unexpected, however, is that some of the emission time traces for larger *L* and higher excitation fluence exhibit very pronounced ringing, resembling the Burnham–Chiao ringing. This contrasts with the assumption of dephasing being too fast at room temperature to allow SF. Instead, ASE should be the dominant process, as concluded before. Ringing in ASE has been previously observed in host–guest systems due to the presence of intermediate states^42^. Moreover, CsPbBr_3_ has been reported to exhibit delayed optical gain due to slow carrier cooling at high excitation fluence^43^. To probe whether the latter could lead to ringing, we performed numerical simulations using a three-level scheme to account for hot carriers (Methods and Supplementary Note 2).

**Extended Data Fig. 8:**
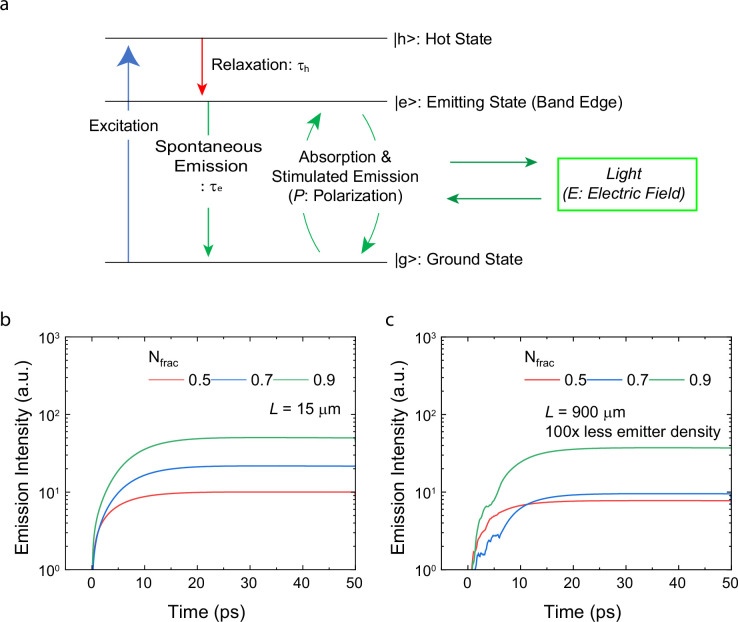
Simulated emission dynamics under stripe excitation. **a**, Schematic illustration of energy levels and relaxation dynamics of the material system modeled in the simulation. **b**, Numerically simulated emission intensity time traces from the high-density thin film for excitation stripe length of 15 µm, showing no signatures of ASE. **c**, Numerically simulated emission intensity time traces from a low-density thin film for excitation stripe length of 900 µm, showing no signatures of ASE.

The simulations (Fig. 5d–f) reproduce not only the emergence of a short ASE pulse for sufficient stripe length and excitation but also the ringing for large *L* and strong excitation. This can be understood by the hot-carrier relaxation continuously filling the emissive band edge state until its population exceeds the ASE threshold and is depleted in a burst of ASE. Repeating this until the population does not reach the ASE threshold any more effectively produces a ringing behaviour. Photon propagation appears to be essential, as for short *L* or low-density films, no ringing is found (Extended Data Fig. 8).

## Conclusions

We present characteristics and transitions between the different collective emission regimes in halide perovskites that pivotally depend on density, temperature and excitation area. At cryogenic temperature, we demonstrated that SF occurs in giant perovskite NCs that are much larger than the exciton Bohr diameter, whereas a transition from typical SF signatures to normal PL (for low-density films) or ASE (for high-density films) was observed at elevated temperatures. Surprisingly, with large excitation beams, a combination of carrier relaxation and propagation effects can mimic SF-like pulse ringing even at room temperature in the ASE regime, suggesting small excitation beams to circumvent similar experimental signatures. Hence, the combined analysis of temporal emission data (decay acceleration, peak growth and ringing) allows extracting information about the coupling in the system, but they always have to be interpreted very carefully together with spectral features and pump geometry (for instance, redshift and excited area size) to allow faithful attribution to the specific emission regime. Our results provide essential understanding and guidelines for the interpretation of peculiar emission dynamics in perovskites, constituting a unique platform to study cooperative phenomena. Furthermore, these can be exploited for the design of ultrabright perovskite light emitting devices such as quantum light sources and lasers/amplifiers.

## Methods

### Synthesis and sample preparation

CsPbBr_3_ NCs were synthesized using the LARP technique following the procedure of ref. ^33^ with slight modifications. The hybrid solution was prepared by mixing solutions of 90 µl of PbBr_2_ (0.67 M in DMF), 465 µl of CsBr (0.043 M in DMF-DMSO in a 1:1 ratio) and 300 µl of OGB (1 M in DMF), where DMF, DMSO and OGB denote dimethyl formamide, dimethyl sulfoxide and oleylguanidinium bromide, respectively. Then, 75 µl of the hybrid solution was injected into a 4-ml vial filled with 2.5 ml of mesitylene under vigorous stirring to initiate the fast nucleation of CsPbBr_3_ NCs. The green solution with bright green luminescence could be observed in 15–20 s. For the purification of CsPbBr_3_ NCs, 0.25 ml of ethyl acetate was added to 0.5 ml of crude solution followed by centrifugation for 4 min at 8 krpm (4,250*g*). The supernatant was discarded, and the precipitate was retained. For preparing low-density NC thin films, the precipitate was redispersed in 0.4 ml of 1% solution of poly(1-vinylpyrrolidone)-*graft*-(1-triacontene) in toluene and 30 µl of the resulting solution of CsPbBr_3_ NCs was drop cast on Si/SiO_2_ substrates and dried under a vacuum. For preparing high-density NC thin films, the precipitate was redispersed in 0.4 ml of toluene and 30 µl of the resulting solution of CsPbBr_3_ NCs was drop cast on Si/SiO_2_ substrates and dried under a vacuum.

### Optical spectroscopy

Time-integrated and time-resolved PL measurements were conducted by mounting the sample in a helium exchange-gas cryostat that operates in a temperature range from 6 K to 300 K. As the excitation source, we used a frequency-doubled regenerative amplifier running at 400 nm with a repetition rate of 1 kHz, delivering pulses with a duration of about 150–250 fs. To prevent parasitic excitation light, short-pass filters (cut-off wavelength, 442 nm) were used. For both excitation and detection, we used the same focusing lens with a 100-mm focal length, resulting in an excitation spot size of about 200 ${\mathrm{\upmu}}{\mathrm{m}}$ in diameter. The recorded PL was spectrally filtered by means of a long-pass filter (cut-off wavelength, 480 nm). For the time-resolved measurements, the emission was dispersed by a 150 lines mm^−1^ grating in a 0.3-m-long monochromator and detected with a streak camera with a nominal time resolution of 2 ps and instrument response function full-width at half-maximum (FWHM) of 4 ps. The time-integrated PL spectra were recorded by a 0.5-m-long spectrograph equipped with a 300 lines mm^−1^ grating and a nitrogen-cooled charge-coupled device camera.

For our experimental conditions, we calculate that an excitation fluence of 1 µJ cm^−2^ leads to the creation of ~44 excitons per NC, which is a fraction of the Mott density and would correspond to 0.15 excitons per NC for 10-nm-sized NCs. In our measurements, at cryogenic temperatures, we excite with fluences in the range of 0.2–14 µJ cm^−2^ and increase up to 640 µJ cm^−2^ at room temperature, which is then far above the Mott density^43^.

With these giant CsPbBr_3_ NCs, up to ten times less excitation fluence compared with formerly reported superlattices of 10-nm NCs^23–25,44^ is needed to achieve the same SF emission acceleration. Presumably, this is due to the higher absorption cross-section at 400 nm, which is supposed to scale linearly with the NC volume^45^, as well as low (in)homogeneous broadening in the giant NCs.

For the measurements that use excitation with a much smaller excitation beam (Extended Data Fig. 6), the laser was focused with a ×100 objective lens (Mitutoyo) to a Gaussian spot diameter with FWHM of 3.5 µm.

For the variable stripe length measurements (Extended Data Fig. 7), the samples were cleaved to allow the light to be collected from the cleaved edge. The stripe-shaped beam spot was obtained by focusing the excitation laser pulse with a cylindrical lens to FWHM of 40 µm × 2,000 µm, with both dimensions well approximated by Gaussians, as confirmed by imaging the excitation spot on a camera. The stripe length was controlled by partially blocking the light with a razor blade on a micrometre stage.

### SF fit model

We conducted curve fitting on the rising signal and the initial decay of the emission time traces with an empirical model function to extract the peak intensity and the time at peak. The model function is a convoluted function of an exponential decay and a Gaussian, that is,

1 $$I\left(t\right)=A\times \exp \left[\frac{{w}^{2}}{4{\tau }^{2}}-\frac{\left(t-{t}_{{\rm{c}}}\right)}{\tau }\right]\times \frac{1}{2}\left(\mathrm{erf}\left[\frac{1}{\sqrt{2}}\left(\frac{t-{t}_{{\rm{c}}}}{w}-\frac{w}{\tau }\right)\right]+1\right).$$

The extracted peak time values are plotted as relative delay time (bottom panels in Supplementary Figs. 7 and 13) by setting the peak time at the weakest excitation fluence as zero for each dataset.

The 1/*e* decay time values were extracted directly from the emission time traces. Further analysis of the obtained decay times was performed with curve fitting with the following model function, which comprises a constant decay for the PL regime and the fluence dependent SF regime above threshold, that is,

2 $$\tau (F\,)=\left\{\begin{array}{l}{\tau }_{\mathrm{Flat}}\left(F\le {F}_{\mathrm{th}}\right)\\ \frac{{\tau }_{0}}{\zeta \times F+1}+{y}_{0}\left(F > {F}_{\mathrm{th}}\right)\end{array}\right.$$

where *F*_th_ is the excitation fluence threshold of emission lifetime shortening. Below the threshold, we use a constant emission lifetime ${\tau }_{{\rm{Flat}}}$, as is expected for PL in a simplified picture without traps or other fluence-dependent quenching. Although, above the threshold, we assumed that the number of coherently coupled dipoles is proportional to the excitation fluence *F* with the proportionality constant *ζ*. *τ*_0_ is fixed to values obtained from the time-resolved PL measurements of NCs at weak excitation fluences (Supplementary Fig. 1). *y*_0_ is inserted to account for effects such as the finite time resolution.

### Computational simulation of ASE

As the excitation energy is much larger than the bandgap energy of the NCs, it has been observed that photoexcited carriers may take several picoseconds to relax into the band edge state^43,46^. Therefore, excitation energy well above the bandgap causes an effective delay in the pumping process from the ground state to the band edge state, that is, the emitting state. Moreover, it is known that the competition between stimulated emission and a delayed pumping process can lead to the ringing of ASE emission pulses^42^. We simulated the ASE dynamics under variable stripe length excitation. First, the propagation of electromagnetic waves in media is described by the following Maxwell’s equations:

3 $$\nabla \times {\bf{E}}=-{\mu }_{0}\frac{\partial \bf{H}}{\partial t},\nabla \times {\bf{H}}={\varepsilon }_{0}\frac{\partial {\bf{E}}}{\partial t}+\frac{\partial {\bf{P}}}{\partial t}.$$

Here **E**(*t, x*) and **H**(*t*, *x*) are the electric and magnetic fields with the electric and magnetic constants *ε*_0_ and *μ*_0_, respectively. The polarization **P**(*t*, *x*) represents the sum of classical oscillator dipoles in the medium. For simplicity, we only considered the propagation of a transverse-electromagnetic wave in the *x* direction.

To replicate the delayed pumping and emission process of the perovskite material, we used a three-level system as the medium. Carriers are excited from the ground state to the high-energy state and then relax to the band edge state non-radiatively. A photon is emitted when a carrier relaxes from the band edge state to the ground state. This system is described using the following pair of rate equations^1^:

4 $$\frac{{\rm{d}}{N}_{{\rm{h}}}}{{\rm{d}}{t}}=-\frac{1}{{\tau }_{{\rm{h}}}}{N}_{{\rm{h}}},$$

5 $$\frac{{\rm{d}}{N}_{{\rm{e}}}}{{\rm{d}}t}=\frac{1}{{\tau }_{{\rm{h}}}}{N}_{{\rm{h}}}-\frac{1}{{\tau }_{{\rm{e}}}}{N}_{{\rm{e}}}+\frac{1}{\hslash \omega }E\frac{{\rm{d}}P}{{\rm{d}}t},$$

6 $$\frac{{\rm{d}}{N}_{{\rm{g}}}}{{\rm{d}}{t}}=\frac{1}{{\tau }_{{\rm{e}}}}{N}_{{\rm{e}}}-\frac{1}{\hslash \omega }E \frac{{\rm{d}}{P}}{{\rm{d}}{t}}.$$

*N*(*t*, *x*) is the number density of carriers at a given time *t* and position *x*. The subscripts h, e and g correspond to the high-energy state, emitting band edge state and ground state, respectively. *τ*_h_ is the time constant of the hot-carrier relaxation process from the high-energy state to the band edge state, and *τ*_e_ is the spontaneous emission lifetime from the band edge state to the ground state. *ħω* corresponds to the energy separation between the band edge state and the ground state.

According to the Lorentz model, the interaction between the electric field and polarization in the media can be described using the following equation:

7 $$\frac{{{\rm{d}}}^{2}P}{{\rm{d}}{t}^{2}}+\Delta \omega \frac{{\rm{d}}P}{{\rm{d}}t}+{\omega }^{2}P=\kappa \Delta {NE}.$$

Δ*ω* is the linewidth of the transition between the band edge state and ground state. Here *κ* = *e*^*2*^*/m*, where *e* is the elementary charge and *m* is the reduced mass of the electron. Δ*N* is the population difference and defined by Δ*N*(*t*, *x*) = *N*_g_(*t*, *x*) – *N*_e_(*t*, *x*).

Since ASE is initiated by spontaneous emission, we introduced an artificial source of the electric field to simulate spontaneous emission numerically^47^. The artificial source is described as follows:

8 $${E}_{\mathrm{source}}\left({t,x}\right)=\sqrt{{N}_{{\mathrm{e}}}\left({t,x}\right){\hslash }\omega \beta \eta } \times \sin \left({\omega t+{\phi }_{\mathrm{random}}}\right).$$

Here *η* is the wave impedance. *β* is a phenomenological parameter, which represents the fraction of photons that is spontaneously emitted in the ASE mode, and *β* is set as 0.01 in our simulation. At each given position *x*, the artificial source has an individual random phase *ϕ*_random_.

Figure 5d–f shows the calculated emission intensity time traces. The total number density of the emitters in the system was set as *N*_total_ = 10^18 ^cm^−3^, and *N*_frac_ indicates the fraction of the excited emitters to *N*_total_. The calculation qualitatively reproduced the experimental results. First, when the system reaches population inversion, the time traces show a fast emission burst because of ASE. The emission burst peak intensity increases with the excitation stripe length because the emitted light is increasingly amplified for a longer excitation stripe length. Besides, the ringing behaviour of the emission pulse is reproduced well in the calculation. It shows that pulse ringing in the time domain is not necessarily a unique signature of SF, but can also occur with ASE due to a combination of relaxation and retardation. On the basis of the similarities of the experimental and calculation results, we conclude that the observed emission dynamics at 300 K can be explained only with ASE, also because for small excitation spots, no acceleration or ringing can be observed at all. The ASE behaviour is expected when dephasing is much faster than SF timescales, as is anticipated at room temperature.

The set of rate equations was solved numerically using Euler’s method. The excitation from the ground state to the high-energy state was modelled as an impulsive response, and the spatial profile was homogeneous all over the stripe-shaped excitation beam spot. The temporal and spatial calculation step sizes were set as Δ*t* = 1 × 10^−16 ^s and Δ*x* = 3 × 10^−8 ^m. The parameters were set as *τ*_e_ = 6 ns and *τ*_h_ = 5 ps. The value of *τ*_e_ was obtained from the emission lifetime measurements at 300 K under a weak excitation fluence (Supplementary Fig. 1h). As for *τ*_h_, we took a value from the typical timescale of the hot-carrier relaxation in CsPbBr_3_ (ref. ^43^). Even though it is known that hot-carrier relaxation times in lead halide perovskites depend on the excitation fluence, we use a constant value in our calculation for simplicity.

For simulation of the transmission and amplified spectra (Extended Data Fig. 3), the commercial finite-difference time-domain software Lumerical Inc. FDTD (Version 25R1) was used. The material dispersion is modelled using multioscillator Lorentz models by fitting the spectroscopically obtained spectra as discussed in the figure caption, using positive and negative imaginary permittivities for absorption and gain, respectively.

## Online content

Any methods, additional references, Nature Portfolio reporting summaries, source data, extended data, supplementary information, acknowledgements, peer review information; details of author contributions and competing interests; and statements of data and code availability are available at 10.1038/s41563-026-02544-3.

## Supplementary information

Supplementary Information Supplementary Notes 1 and 2, Figs. 1–13 and References.

## Data Availability

The data supporting the findings of this study are available via Zenodo at 10.5281/zenodo.18609757 (ref. ^48^).
