# Supplementary material for: Transition between cooperative emission regimes in giant perovskite nanocrystals
Source: Nat Mater. 2026 Mar 31;25(7):1146–53. doi: 10.1038/s41563-026-02544-3 (PMC13323054; doi:10.1038/s41563-026-02544-3)
Supplement: Supplementary file 1 — Supplementary Notes 1 and 2, Figs. 1–13 and References. [file 41563_2026_2544_MOESM1_ESM.pdf]

nature materials

Supplementary information https://doi.org/10.1038/s41563-026-02544-3

Transition between cooperative emission regimes in giant perovskite nanocrystals

In the format provided by the authors and unedited

Table of Contents

Supplementary Note 1: Key characteristics of superfluorescence

Supplementary Note 2: Emission pulse ringing behavior in ASE regime

Fig. S1 | Spectrally integrated PL time traces of the low-density thin film at different temperatures under weak excitation.

Fig. S2 | Emission spectra of the low-density thin film under increasing excitation fluence at different temperatures.

Fig. S3 | Time-integrated emission intensity as a function of excitation fluence of the low-density thin film at different temperatures.

Fig. S4 | Peak wavelength of emission spectra of the low-density thin film under increasing excitation fluence at different temperatures.

Fig. S5 | Emission peak widths (FWHM) of the low-density thin film under increasing excitation fluence at different temperatures.

Fig. S6 | Spectrally integrated emission time traces of the low-density thin film with increasing excitation fluence at different temperatures.

Fig. S7 | Emission dynamics parameters from the low-density thin film as a function of the excitation fluence and the model curves (solid lines) of: (top) 1/e emission decay time; (middle) time-resolved emission peak intensity; (bottom) emission pulse build-up time.

Fig. S8 | Emission spectra of the high-density thin film under increasing excitation fluence at different temperatures.

Fig. S9 | Time-integrated emission intensity of the high-density thin film at different temperatures.

Fig. S10 | Peak wavelength of emission spectra of the high-density thin film at different temperatures.

Fig. S11 | Emission peak widths (FWHM) of the high-density thin film at different temperatures.

Fig. S12 | Spectrally integrated emission time traces of the high-density thin film with increasing excitation fluence at different temperatures.

Fig. S13 | Emission dynamics parameters of the high-density thin film as a function of the excitation fluence and the model curves (solid lines) of: (top) 1/e emission decay time; (middle) time-resolved emission peak intensity; (bottom) emission pulse build-up time.

References

Supplementary Note 1: Key characteristics of superfluorescence

As many theoretical and experimental papers have reported, SF exhibits several key characteristics in the emission dynamics. First, SF features an accelerated decay with a radiative lifetime $\tau_R \propto \tau_0/N$, where τ_0 is the single-emitter radiative lifetime and N is the number of excited coupled emitters; second, the SF pulse peak intensity grows as $I_{peak} \propto N^2$; third, due to the time that the emitters need to spontaneously synchronize, SF emission occurs with a finite delay time after excitation that follows $\tau_D \propto (\ln N)/N$; fourth, the so-called Burnham-Chiao ringing can arise from Rabi-like periodic energy exchange between the electromagnetic field and the macroscopic polarization in the material[1,2] or coherent reabsorption/reemission during propagation; fifth, from a quantum optics viewpoint, SF pulses exhibit photon bunching, and a controlled number of excited emitters could be used to realize strongly correlated multi-photon states[3]. These features are important signatures in experiments to distinguish SF from other emission regimes such as ASE, with the caveat that many of these features can also arise in a similar form from different mechanisms than SF, always requiring multiple dynamical signatures and spectral properties to be taken in conjunction in order to attribute the emission regime.

Supplementary Note 2: Emission pulse ringing behavior in ASE regime

As shown in Fig. 5, we observed pronounced emission pulse ringing in stripe excitation measurements at room temperature. The ringing in emission time traces resembles the Burnham-Chiao ringing, which is one signature of SF. However, it contradicts with our assumption from the measurements with smaller beam and normal-incidence detection (Fig. 4): Dephasing and decoherence are too fast at room temperature to allow SF, and ASE should be the dominant process instead. Indeed, ringing can also manifest in an ASE process, previously observed in host-guest systems and ascribed to the presence of intermediate states[4]. Furthermore, $CsPbBr_3$ has been reported to exhibit delayed optical gain due to slow carrier cooling at high excitation fluence[5]. To probe whether the latter could indeed give rise to an ASE ringing behavior, we performed numerical simulations using a three-level scheme to account for hot carriers (see Methods) (Fig. 5d-f).

The simulations reproduce the emergence of a short emission peak, which corresponds to ASE, and which keeps getting more pronounced by increasing L or/and N_{frac}. Notably, like in the experiments, ASE could not be observed for small excitation beams or for low-density thin films (Extended Data Fig. 8). Moreover, the simulation also replicates the pulse ringing for large L and N_{frac}. This can be understood by the hot-carrier relaxation continuously filling the emissive band edge state, until its population exceeds the ASE threshold and is depleted in a burst of ASE. Subsequently, it is replenished by relaxing hot carriers and eventually reaches again the ASE regime, effectively producing a ringing behavior. Hence, photon propagation and its concomitant retardation are playing a role for the observed emission dynamics, but not for short L where no ringing is observed. Besides, the absence of the ringing behavior in normal incident excitation (Fig. 4f), can be attributed to the transverse effects during the light propagation[6].

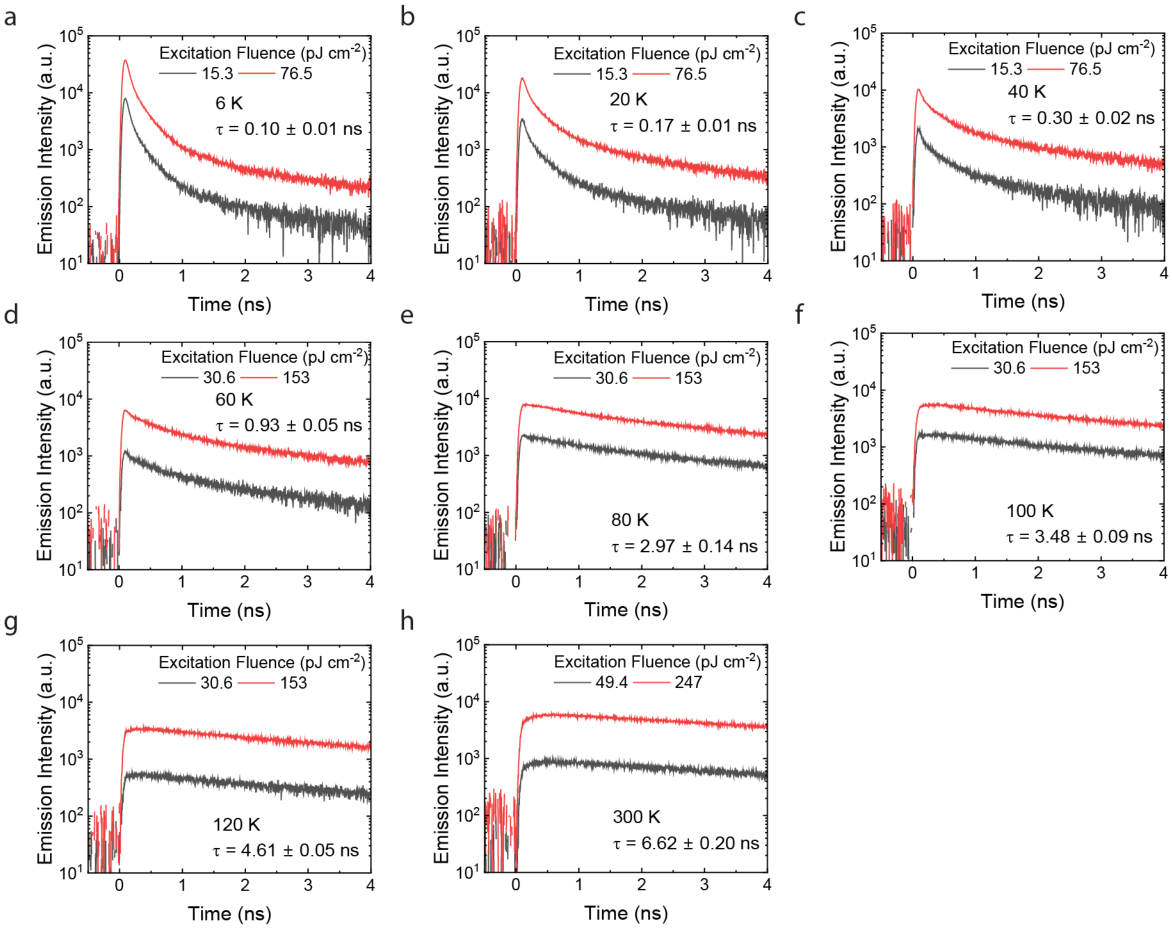

Fig. S1 | Spectrally integrated PL time traces of the low-density thin film at different temperatures under weak excitation. The sample temperatures are 6 K (a), 20 K (b), 40 K (c), 60 K (d), 80 K (e), 100 K (f), 120 K (g), and 300 K (h). τ indicates the 1/e emission decay time at each temperature. At 120 K and 300 K the decay time was obtained by curve fitting with a single exponential function since the 1/e decay times are longer than the measurement time window. The emission lifetimes obtained under this weak excitation fluence are taken as the basic emitter decay time τ_0 in the further analysis.

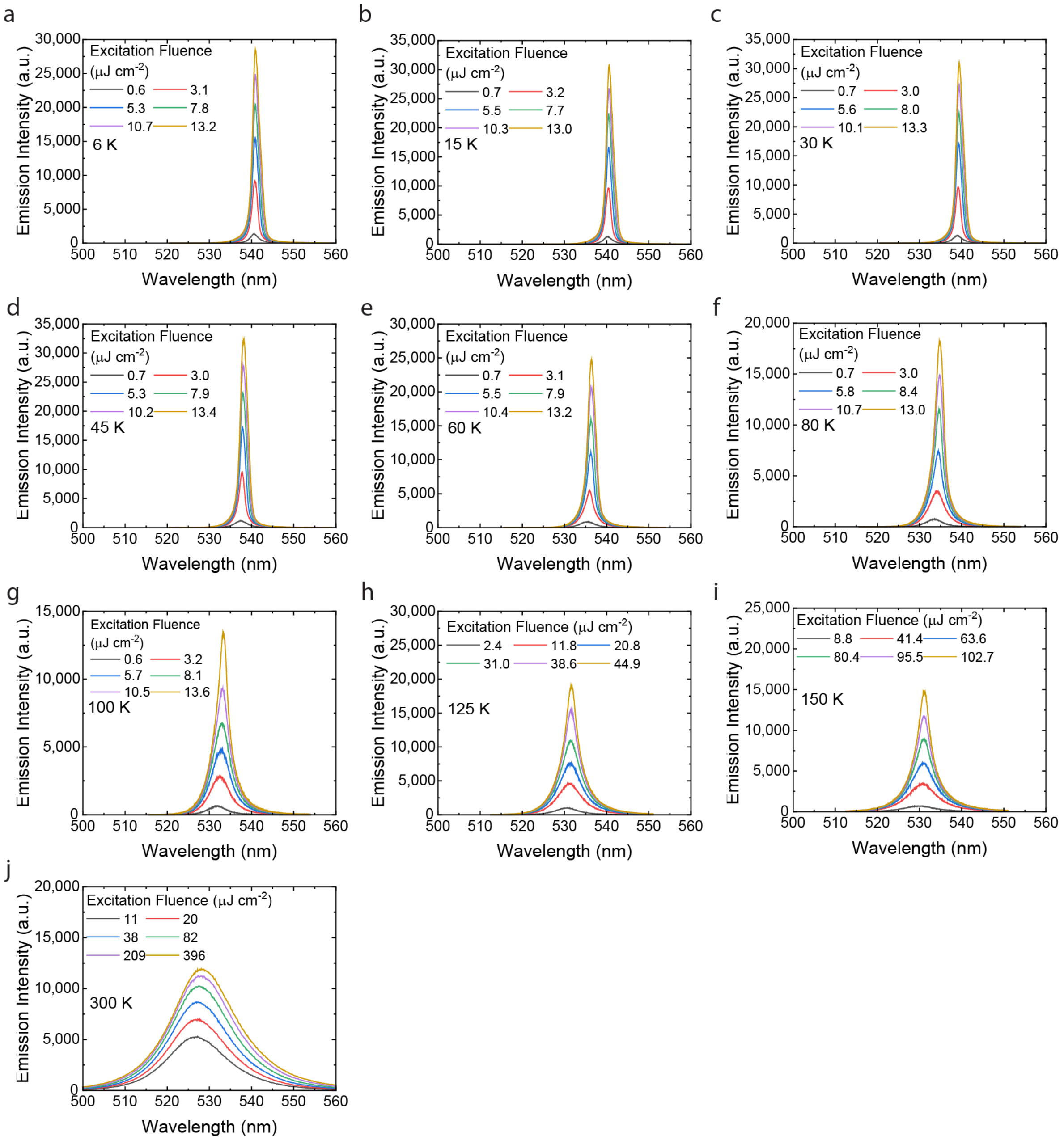

Fig. S2 | Emission spectra of the low-density thin film under increasing excitation fluence at different temperatures. The sample temperatures are 6 K (a), 15 K (b), 30 K (c), 45 K (d), 60 K (e), 80 K (f), 100 K (g), 125 K (h), 150 K (i), and 300 K (j).

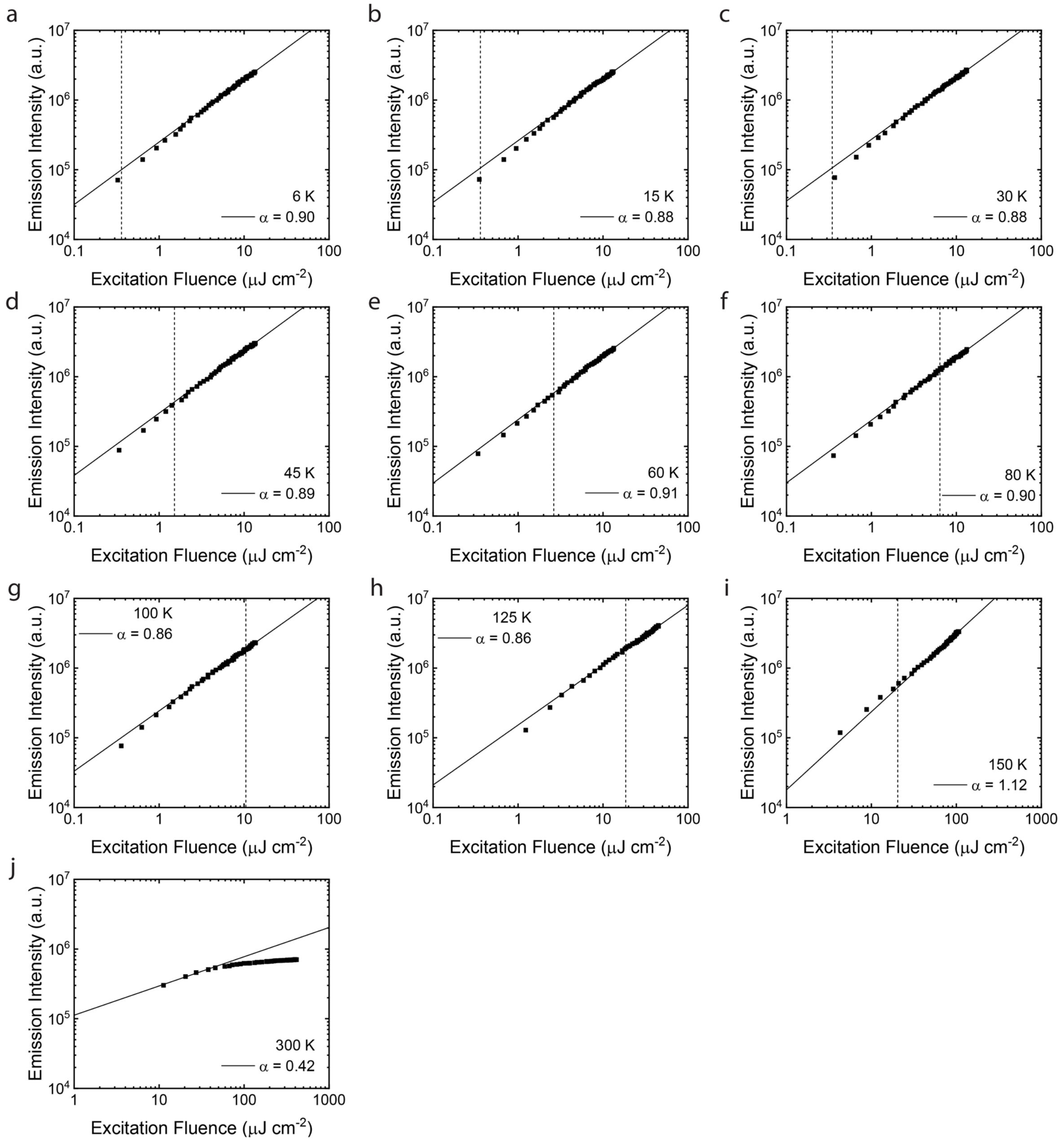

Fig. S3 | Time-integrated emission intensity as a function of excitation fluence of the low-density thin film at different temperatures. The sample temperatures are 6 K (a), 15 K (b), 30 K (c), 45 K (d), 60 K (e), 80 K (f), 100 K (g), 125 K (h), 150 K (i), and 300 K (j). α indicates the exponent value obtained from a power-law fit (solid line). The dashed lines indicate the threshold of emission 1/e decay lifetime shortening obtained from the analysis shown in Fig. S7. The emission intensity increases almost linearly with excitation fluence in the range 6 – 150 K, suggesting the absence of nonradiative exciton-density-dependent quenching. However, upon approaching room temperature, the overall emission intensity reduces significantly, which we attribute to increasingly efficient nonradiative decay processes, consistent with the observed reduction of the PL QY to ~5% at room temperature (Extended Data Fig. 1b).

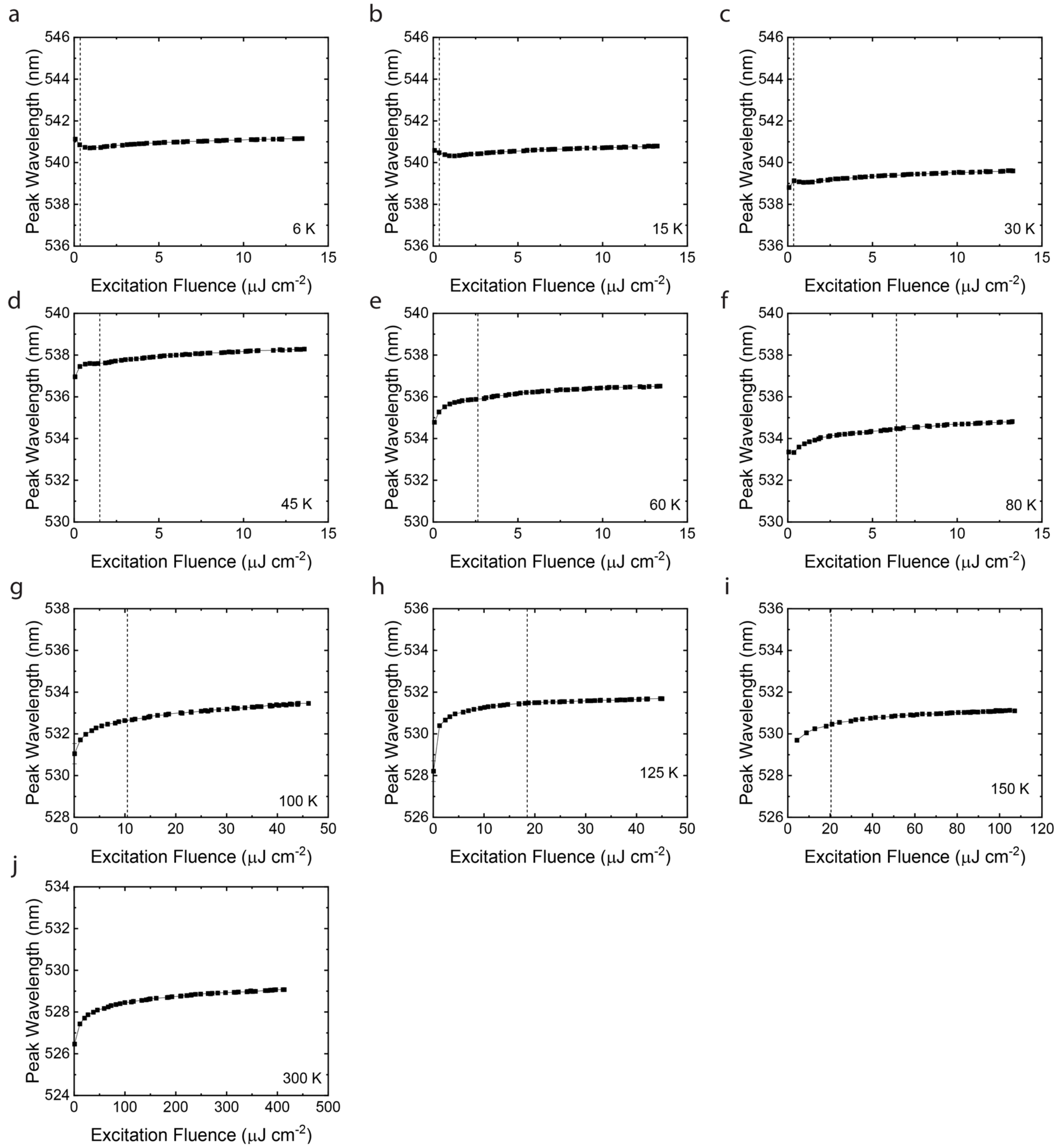

Fig. S4 | Peak wavelength of emission spectra of the low-density thin film under increasing excitation fluence at different temperatures. The sample temperatures are 6 K (a), 15 K (b), 30 K (c), 45 K (d), 60 K (e), 80 K (f), 100 K (g), 125 K (h), 150 K (i), and 300 K (j). The dashed lines indicate the threshold of emission 1/e decay lifetime shortening obtained from the analysis shown in Fig. S7.

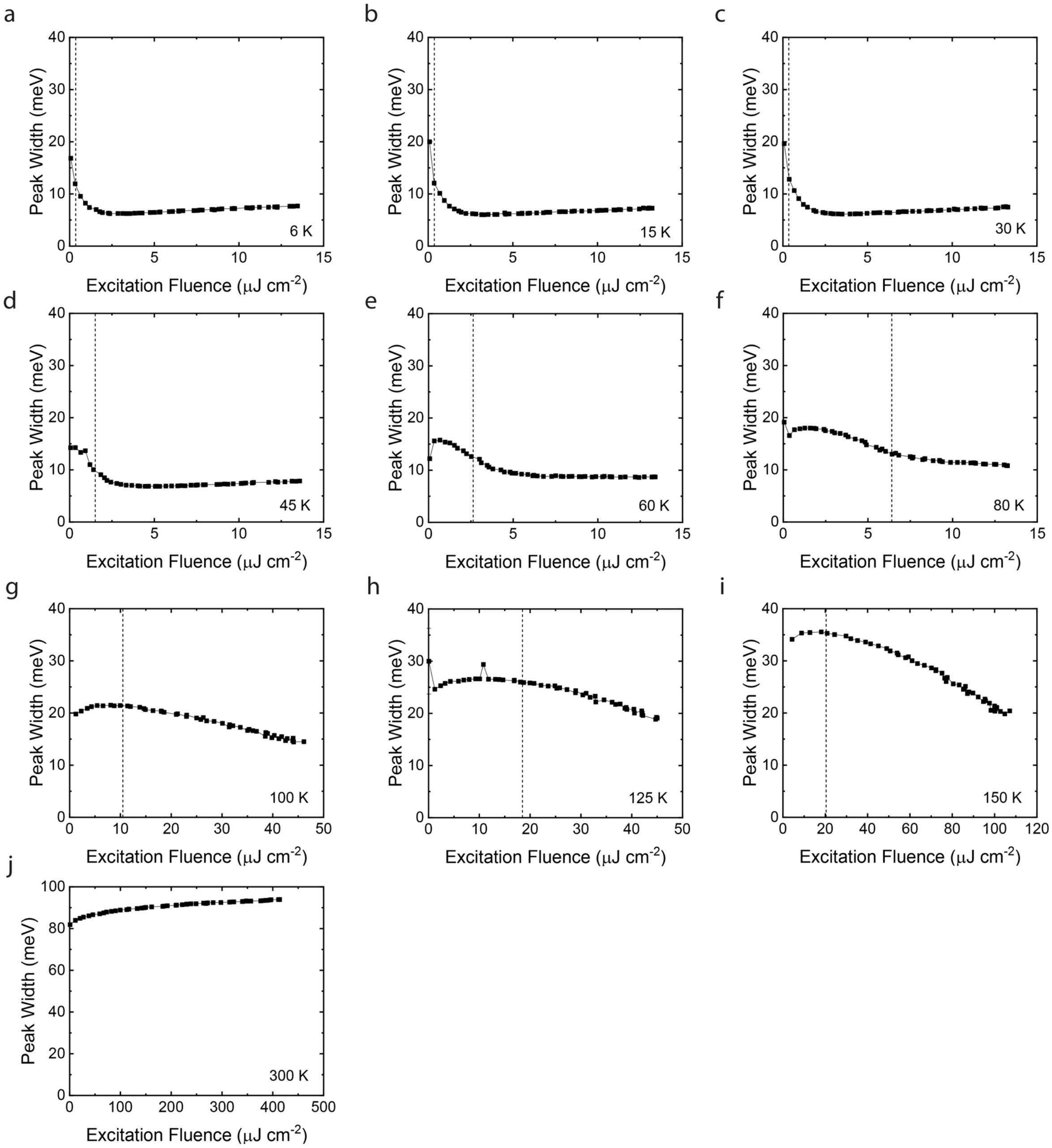

Fig. S5 | Emission peak widths (FWHM) of the low-density thin film under increasing excitation fluence at different temperatures. The sample temperatures are 6 K (a), 15 K (b), 30 K (c), 45 K (d), 60 K (e), 80 K (f), 100 K (g), 125 K (h), 150 K (i), and 300 K (j). The dashed lines indicate the threshold of emission 1/e decay lifetime shortening obtained from the analysis shown in Fig. S7.

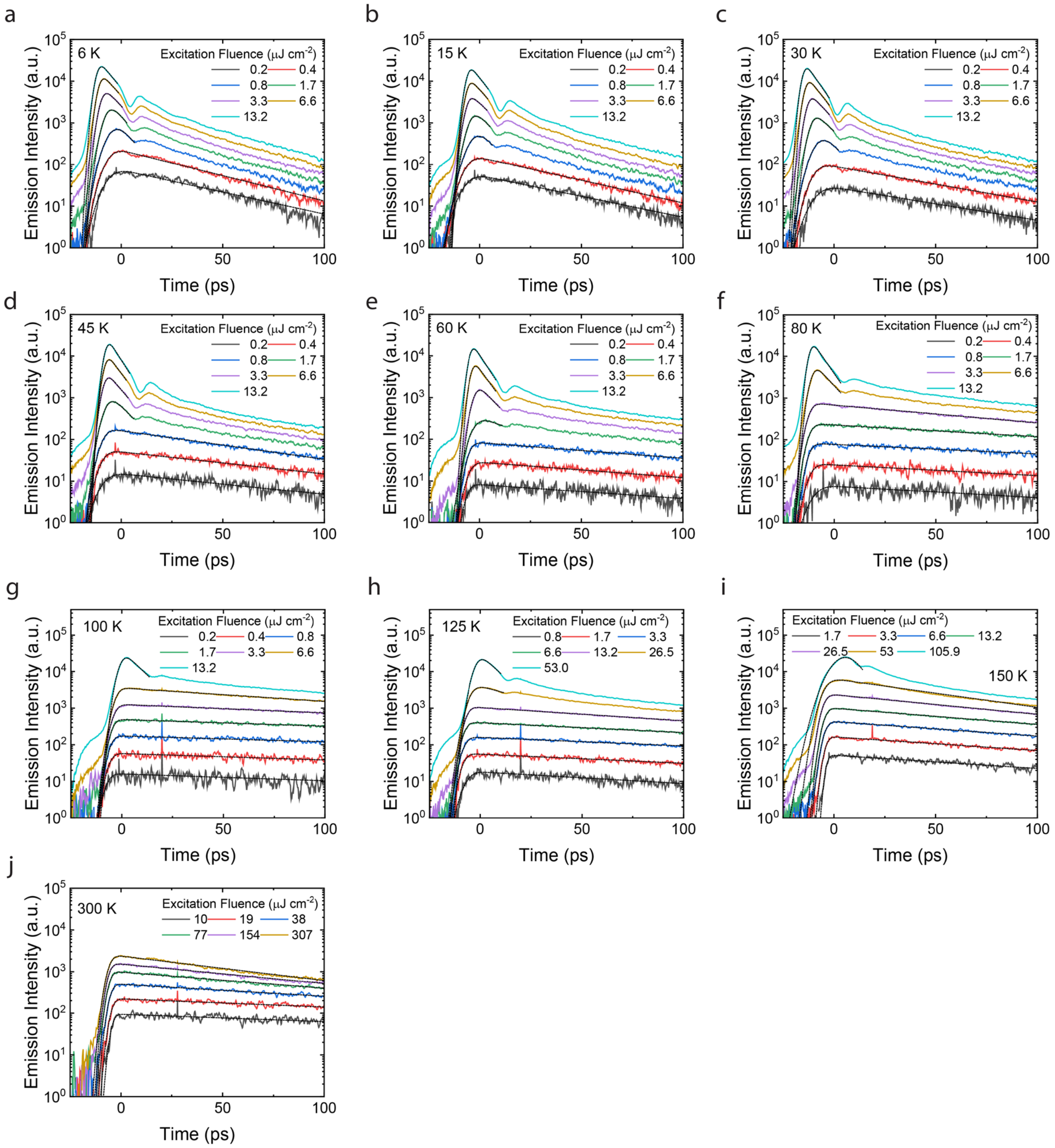

Fig. S6 | Spectrally integrated emission time traces of the low-density thin film with increasing excitation fluence at different temperatures. The sample temperatures are 6 K (a), 15 K (b), 30 K (c), 45 K (d), 60 K (e), 80 K (f), 100 K (g), 125 K (h), 150 K (i), and 300 K (j). The dashed curves are the results of fitting analysis on the signal rising and initial decay.

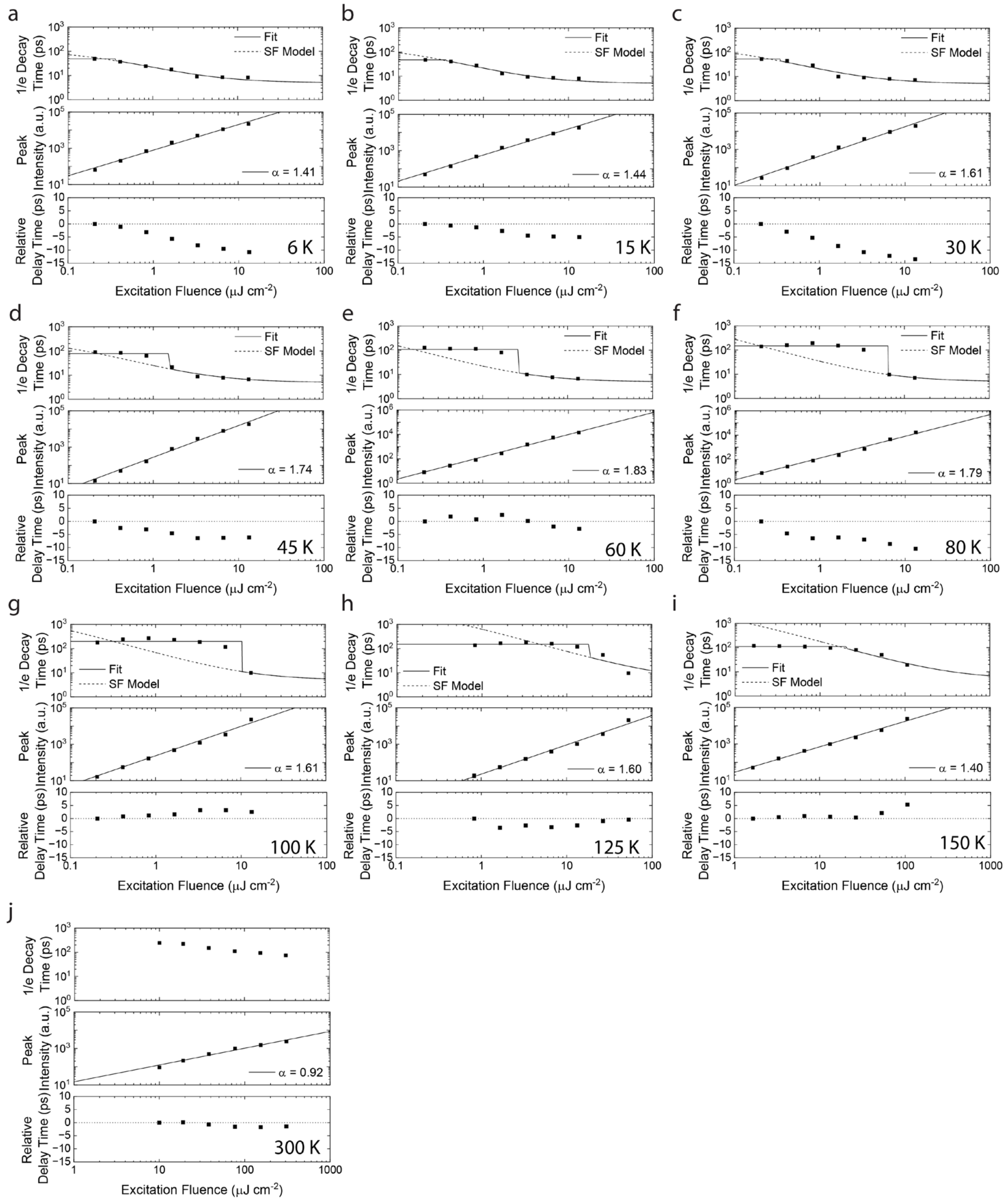

Fig. S7 | Emission dynamics parameters from the low-density thin film as a function of the excitation fluence and the model curves (solid lines) of: (top) 1/e emission decay time; (middle) time-resolved emission peak intensity; (bottom) emission pulse build-up time. The sample temperatures are 6 K (a), 15 K (b), 30 K (c), 45 K (d), 60 K (e), 80 K (f), 100 K (g), 125 K (h), 150 K (i), and 300 K (j). In the top panels, solid curves are the results of fitting analysis with the piecewise model function. The dashed curves are the extrapolated curves of the above-threshold part of the fitted curves. In the middle panels, α indicates the exponent value obtained from a power-law fit (solid lines). In the bottom panels, the time at the emission peak is shown as relative delay time by setting the peak time at the weakest excitation fluence as zero for each dataset. Below 100 K, it is observed that the amount of the relative delay time shortening does not show a monotonic trend over the temperature, for example, the amount of shortening at 6 K is larger than that at 15 K, while at 30 K the amount of delay time shortening is again larger than 15

K. Contributions of various temperature-dependent physical effects could explain these observations. Although dephasing generally hinders SF, it can also have indirect effects in $CsPbBr_3$ NCs: on one hand, it reduces the exciton oscillator strength by diminishing single-photon superradiant enhancement[7], while on the other hand, thermal broadening may alleviate spectral mismatch among inhomogeneous emitters.

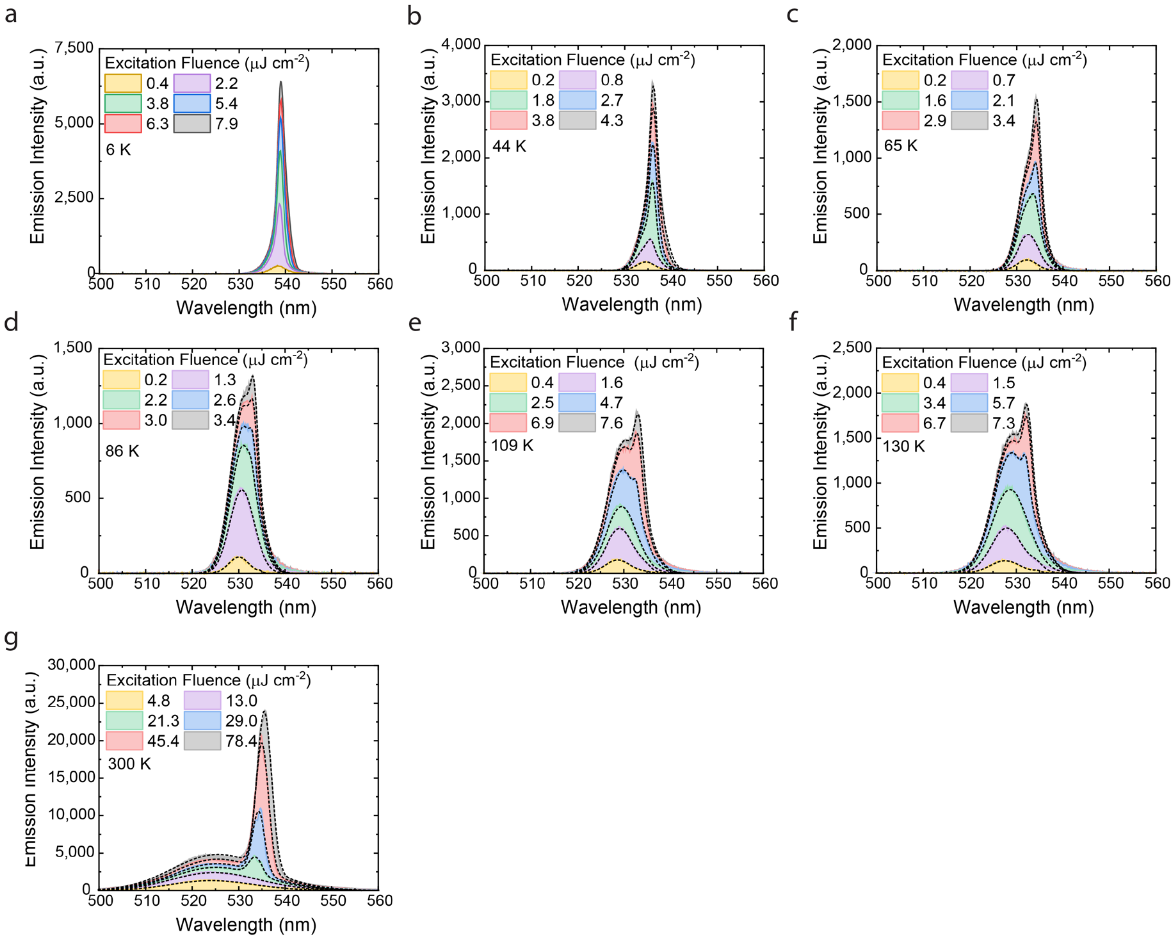

Fig. S8 | Emission spectra of the high-density thin film under increasing excitation fluence at different temperatures. The sample temperatures are 6 K (a), 44 K (b), 65 K (c), 86 K (d), 109 K (e), 130 K (f), and 300 K (g). The dotted curves in (b-g) are the results of curve fitting with a double Gaussian peak function.

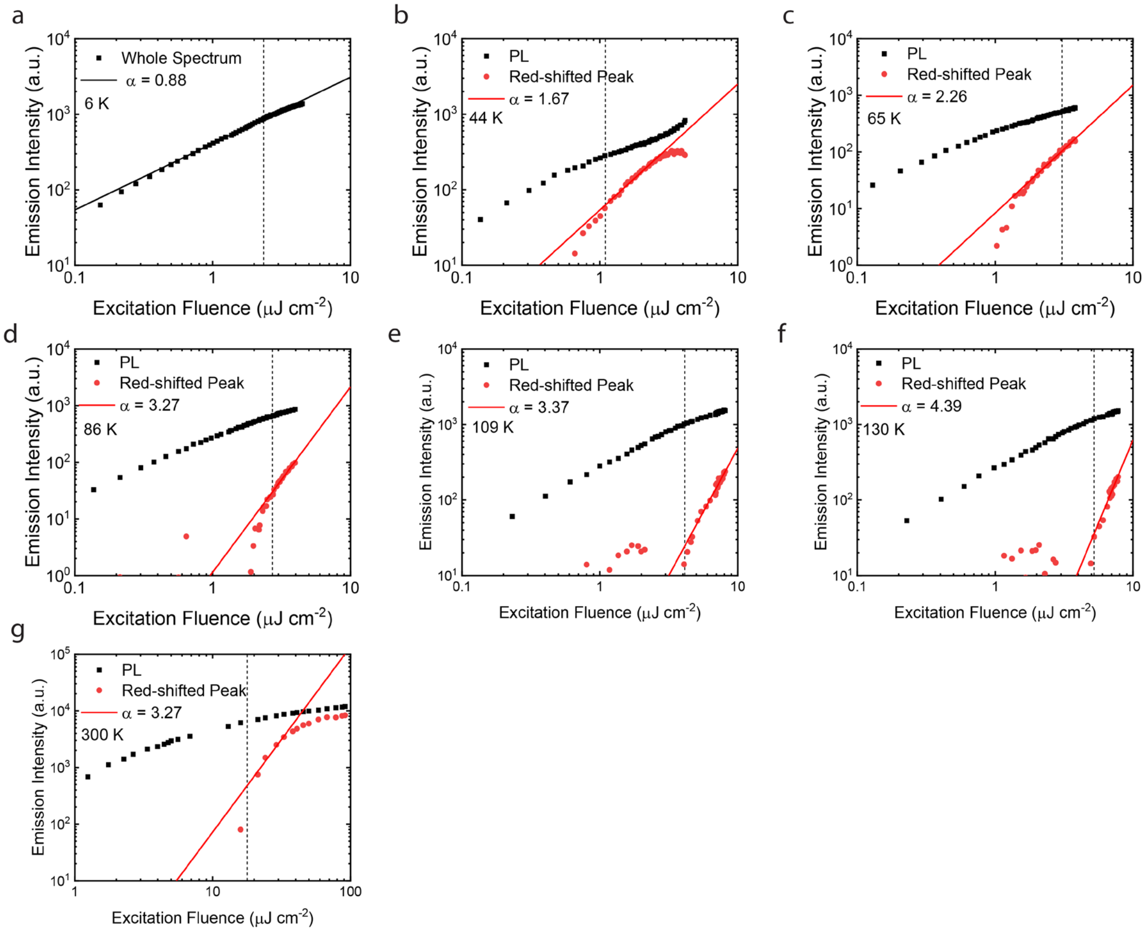

Fig. S9 | Time-integrated emission intensity of the high-density thin film at different temperatures. The sample temperatures are 6 K (a), 44 K (b), 65 K (c), 86 K (d), 109 K (e), 130 K (f), and 300 K (g). The plotted emission intensity in (a) is wavelength- and time-integrated signal intensity of emission spectra. For (b-g), the plotted emission intensities were obtained as the products of the peak amplitudes and the peak widths from the double Gaussian fit shown in Fig. S8. "PL" (black data points) indicates the emission intensity from the broad emission signal, while "Red-shifted Peak" (red data points) indicates the emission peak with smaller linewidth and redshifted peak energy compared to the broad PL signal. α indicates the exponent value obtained from a power-law fit on the redshifted peak signals (solid lines). The dashed lines indicate the threshold of emission 1/e decay lifetime shortening obtained from the analysis shown in Fig. S13.

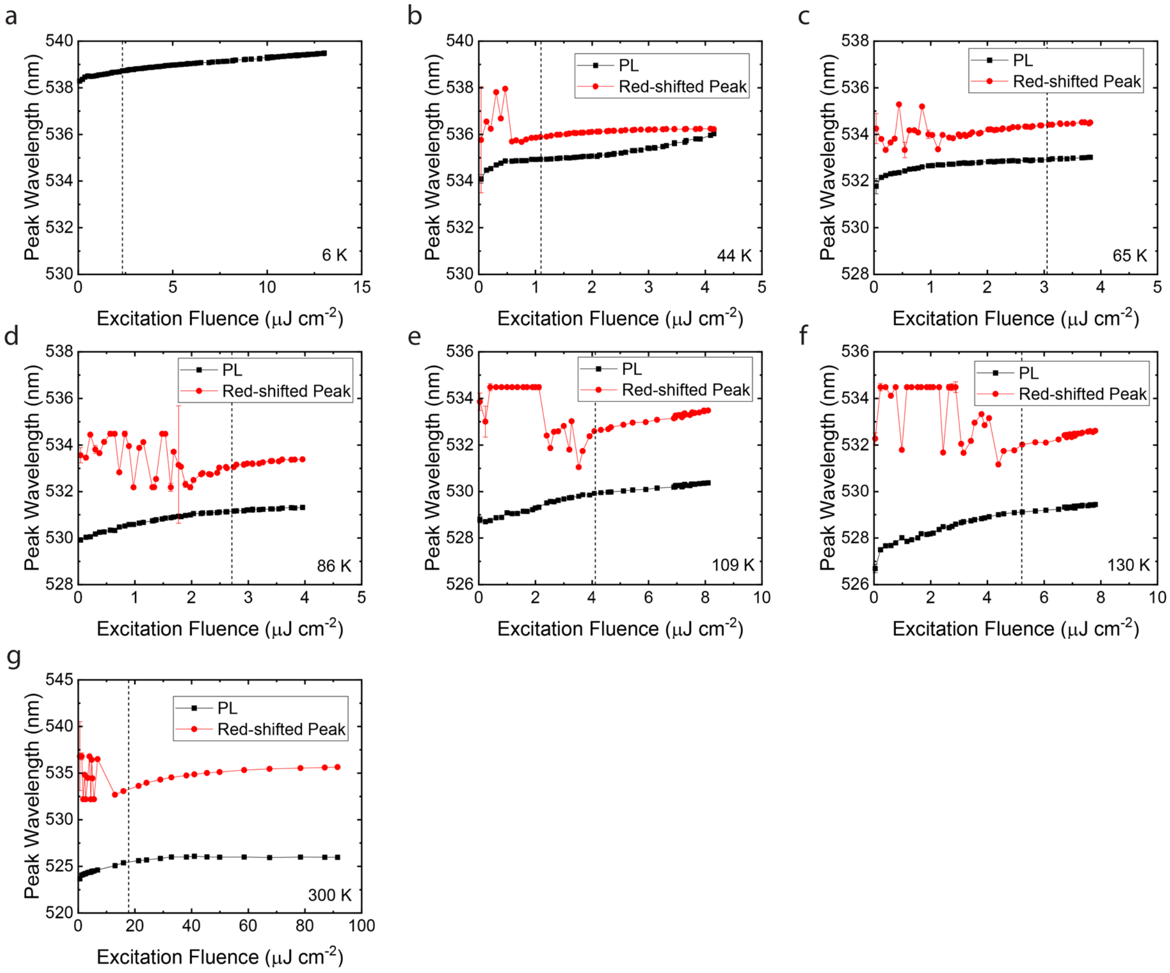

Fig. S10 | Peak wavelength of emission spectra of the high-density thin film at different temperatures. The sample temperatures are 6 K (a), 44 K (b), 65 K (c), 86 K (d), 109 K (e), 130 K (f), and 300 K (g). The plotted emission peak wavelength in (a) is obtained from single Lorentzian fitting on the experimental data. For (b-g), the plotted emission wavelength is obtained from the double Gaussian fit shown in Fig. S8. "PL" (black data points) indicates the peak wavelength of the broad emission signal, while "Red-shifted Peak" (red data points) indicates the peak wavelength of redshifted narrow emission peak. The dashed lines indicate the threshold of emission 1/e decay lifetime shortening obtained from the analysis shown in Fig. S13. Error bars indicate the standard errors of the fit parameters from curve fitting analysis.

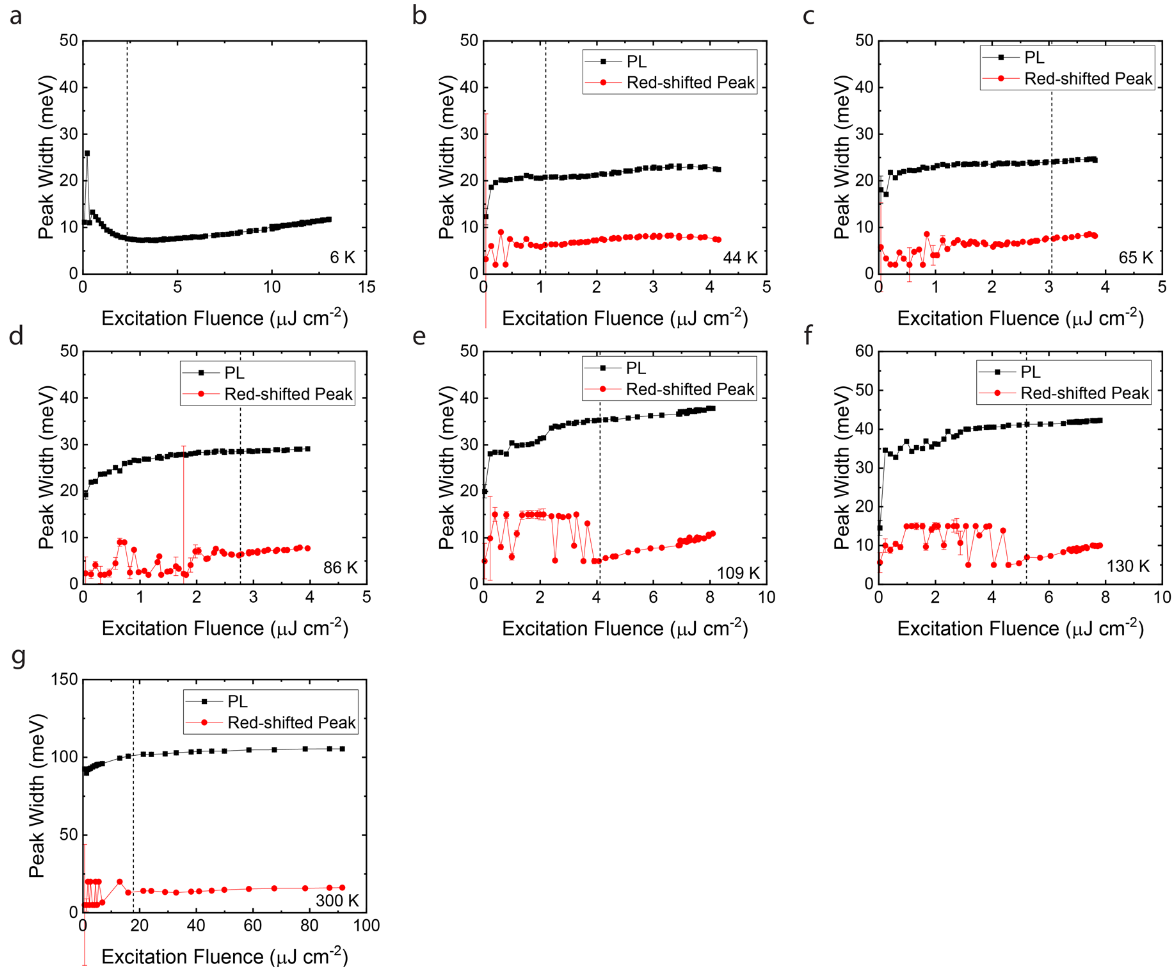

Fig. S11 | Emission peak widths (FWHM) of the high-density thin film at different temperatures. The sample temperatures are 6 K (a), 44 K (b), 65 K (c), 86 K (d), 109 K (e), 130 K (f), and 300 K (g). The plotted emission peak width in (a) is obtained from single Lorentzian fitting on the experimental data. For (b-g), the plotted emission peak widths are obtained from the double Gaussian fit shown in Fig. S8. "PL" (black data points) indicates the peak width of the broad emission signal, while "Red-shifted Peak" (red data points) indicates the width of redshifted narrow emission peak. The dashed lines indicate the threshold of emission 1/e decay lifetime shortening obtained from the analysis shown in Fig. S13. Error bars indicate the standard errors of the fit parameters from curve fitting analysis.

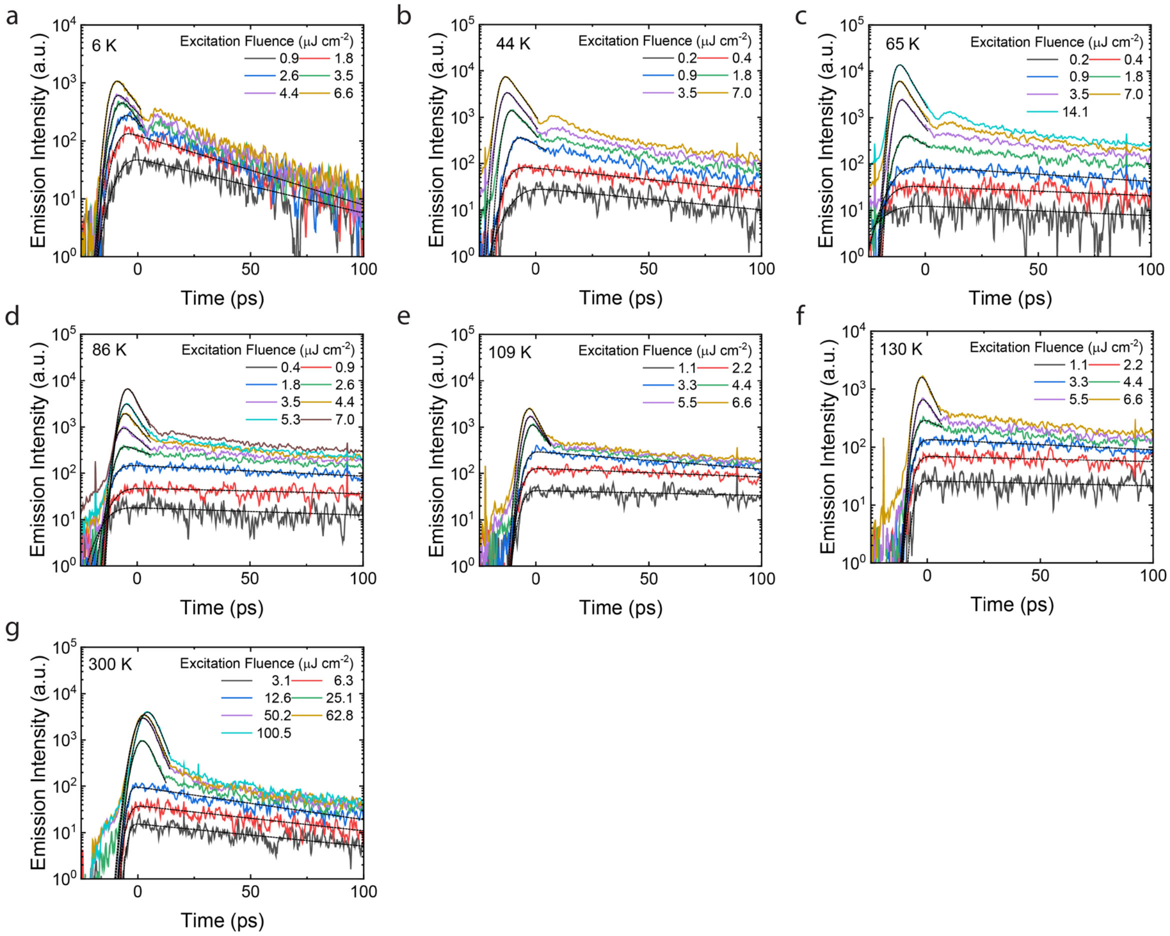

Fig. S12 | Spectrally integrated emission time traces of the high-density thin film with increasing excitation fluence at different temperatures. The sample temperatures are 6 K (a), 44 K (b), 65 K (c), 86 K (d), 109 K (e), 130 K (f), and 300 K (g). The dashed curves are the results of fitting analysis on the signal rising and initial decay.

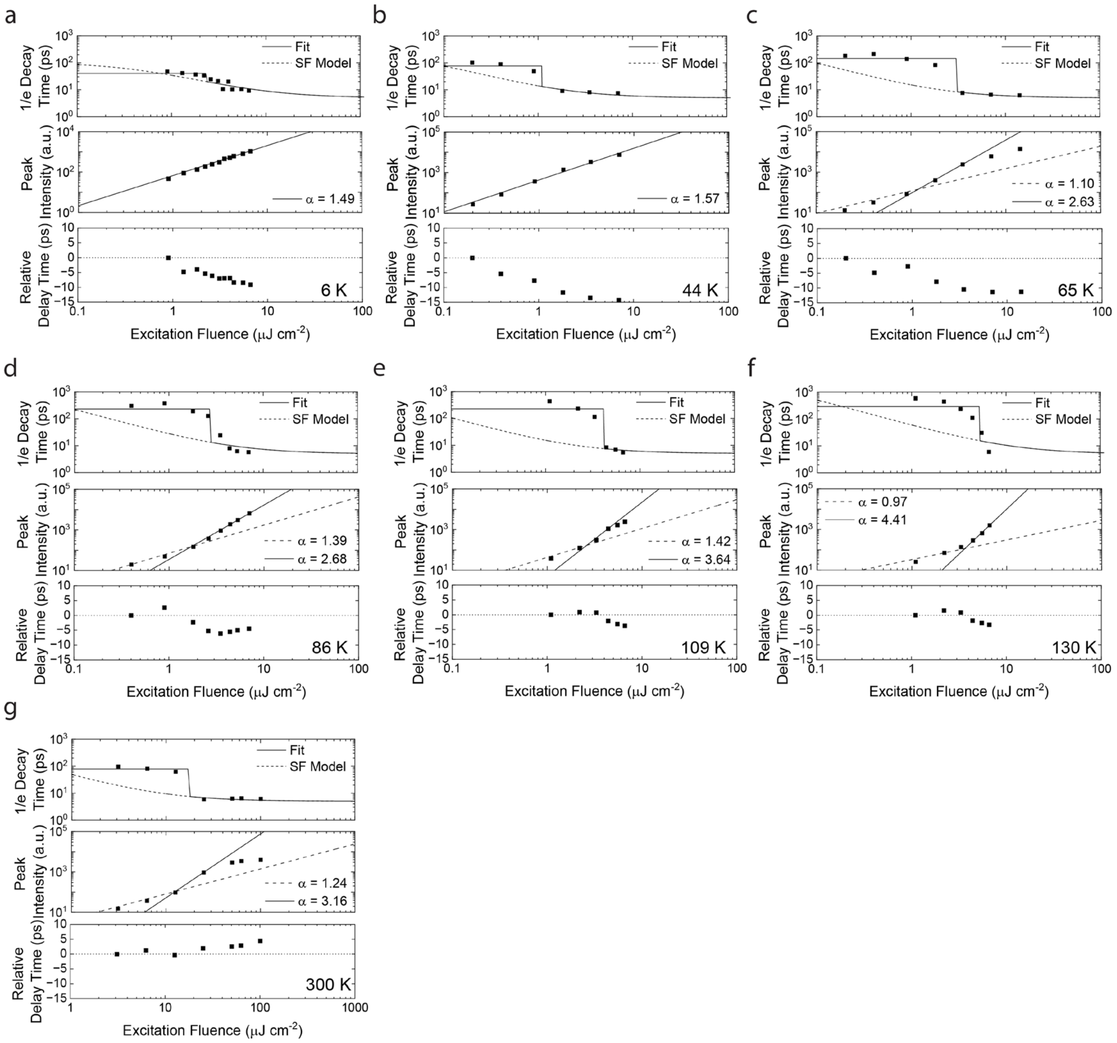

Fig. S13 | Emission dynamics parameters of the high-density thin film as a function of the excitation fluence and the model curves (solid lines) of: (top) $1/e$ emission decay time; (middle) time-resolved emission peak intensity; (bottom) emission pulse build-up time. The sample temperatures are 6 K (a), 44 K (b), 65 K (c), 86 K (d), 109 K (e), 130 K (f), and 300 K (g). In the top panels, solid curves are the results of fitting analysis with the piecewise model function. The dashed curves are the extrapolated curves of the above-threshold part of the fitted curves. In the middle panels, α indicates the exponent value obtained from a power-law fit (solid and dashed lines). In the bottom panels, the time at the emission peak is shown as relative delay time by setting the peak time at the weakest excitation fluence as zero for each dataset.

References

1. Burnham, D. C. & Chiao, R. Y. Coherent Resonance Fluorescence Excited by Short Light Pulses. *Phys. Rev.* **188**, 667–675 (1969).
2. Heinzen, D. J., Thomas, J. E. & Feld, M. S. Coherent Ringing in Superfluorescence. *Phys. Rev. Lett.* **54**, 677–680 (1985).
3. Muñoz, C. S. *et al.* Emitters of N-photon bundles. *Nat. Photonics* **8**, 550–555 (2014).
4. Kallinger, C. *et al.* Picosecond amplified spontaneous emission bursts from a molecularly doped organic semiconductor. *J. Appl. Phys.* **91**, 6367 (2002).
5. Schlaus, A. P. *et al.* How lasing happens in CsPbBr3 perovskite nanowires. *Nat. Commun.* **10**, 265 (2019).
6. Mattar, F. P., Gibbs, H. M., McCall, S. L. & Feld, M. S. Transverse Effects in Superfluorescence. *Phys. Rev. Lett.* **46**, 1123–1126 (1981).
7. Zhu, C. *et al.* Single-photon superradiance in individual caesium lead halide quantum dots. *Nature* **626**, 535–541 (2024).
